# Research on Quality Evaluation of the Seeds of *Cichorium glandulosum Boiss. et Huet.*

**DOI:** 10.3390/foods14081434

**Published:** 2025-04-21

**Authors:** Xu Chen, Jianshuang Jiang, Fengling Li, Wen Lei, Juan Li, Xiaoting Wang, Ayiben Wenhua, Jingjing Xia, Jiang He

**Affiliations:** 1 Key Laboratory of Uygur Medicine, Xinjiang Institute of Materia Medica, No. 140, Xinhua North Road, Tianshan District, Urumqi 830004, China; 2 School of Pharmaceutical Sciences and Institute of Materia Medica, Xinjiang University, Urumqi 830046, China; 3 State Key Laboratory of Bioactive Substance and Function of Natural Medicines, Institute of Materia Medica, Peking Union Medical College, Chinese Academy of Medical Sciences, Beijing 100050, China

**Keywords:** *Cichorium glandulosum Boiss. et Huet.*, quality evaluation, UHPLC-MS/MS, infrared spectroscopy, chemometrics

## Abstract

The seeds of *Cichorium glandulosum Boiss. et Huet.* (CS) are known for various effects. However, the research on the establishment of quality evaluation standards for CS is currently limited. Therefore, this study employed Ultra Performance Liquid Chromatography–Tandem Mass Spectrometry (UPLC-MS/MS) to analyze the components of CS. Forty-nine compounds were identified through manual analysis and database comparison. The components were then verified using HPLC and standards. Additionally, 19 batches were collected to establish the fingerprint chromatogram. Five major chemical components were selected for subsequent analysis. MIR, combined with three variable selection algorithms and three preprocessing methods, was used to build prediction models. For the three indexes of Chlorogenic Acid, 1,4-Dicaffeoylquinic Acid, and 1,5-Dicaffeoylquinic Acid, the R^2^ values for both the training set and test set were above 0.9, the RPD values were all greater than 2.5, and the RER values were greater than 10. This indicated that the combination of mid-infrared spectroscopy and chemometrics had excellent model applicability and prediction performance for these three indexes. A quality evaluation system has been initially established, laying a foundation for research on quality evaluation of CS.

## 1. Introduction

*Cichorium glandulosum Boiss. et Huet.* is mainly distributed in the Xinjiang Uyghur Autonomous Region of China, as well as in Turkey and the Caucasus region [1,2,3]. The seeds of *Cichorium glandulosum Boiss. et Huet.* (CS) are commonly used as edible and medicinal materials by ethnic groups in Pakistan, India, and the western regions of China [1,2]. In addition, CS is widely utilized in traditional Uighur medical prescriptions, such as Compound Muniziqi Granules, Liver-Protecting Buzure Granules, and Dinar Syrup, and has various pharmacological effects, including liver protection, anti-inflammatory properties, and improvement of abnormal body fluids [4,5,6,7,8]. Although CS is widely applied, there is a lack of standards for its quality evaluation. Only the roots and above-ground parts of *Cichorium glandulosum Boiss. et Huet.* are included in the Chinese Pharmacopoeia [9]. Additionally, the “Xinjiang Uighur Autonomous Region Uighur Medicinal Materials Standard” identifies CS based solely on their appearance and microscopic characteristics [10].

The primary methods currently employed for predicting quality markers in traditional Chinese medicine (TCM) encompass inductive summarization, chemical component analysis via fingerprint chromatogram, network pharmacology, prediction of component-disease targets through molecular docking, and pharmacological experimentation [11,12,13]. For instance, Li et al. conducted an analysis to determine the compositional differences between various types and parts of chicory. This was achieved through comparative screening of fingerprint chromatogram, network pharmacology, molecular docking, and cell experiments. As a result, chicory acid and lactucopicrin were identified as quality markers for chicory, which are significant in the treatment of gout and in reducing uric acid [14]. Tao et al. have reviewed the chemical constituents and modern pharmacological research of *Stephania tetrandra* and performed predictive analysis on its quality markers from three aspects: unique chemical constituents, the pharmacodynamic substance basis of pharmacological activities, and network pharmacology, providing a scientific basis for the quality control of *Stephania tetrandra* [15]. Cheng et al. established an analysis method for the fingerprint of *Anemarrhenae Rhizoma* and, by combining network pharmacology and molecular docking technology, discovered that three active components of *Anemarrhenae Rhizoma* were closely related to its efficacy attributes, providing a reference for the quality control and laying the foundation for exploring its anti-inflammatory mechanism [16]. Research on TCM based on quality markers is rapidly increasing, providing insights into the development of quality standards and quality control of traditional ethnic medicines.

Infrared spectroscopy (IR), which provides abundant functional group information, combined with chemometrics, has been widely applied in the field of TCM, including the identification and analysis of TCM, processed TCM, Chinese patent medicines, and so on [17]. For example, Bertol, G. et al. utilized Mid-infrared spectroscopy (MIR) combined with chemometrics to identify *Mikania glomerata Spreng.* and *M. laevigata Sch. Bip. ex Baker* [18]. Guo, YZ et al. applied the macroscopic infrared fingerprinting method, including MIR, second derivative infrared spectroscopy (SD-IR), and two-dimensional correlation infrared spectroscopy (2D-IR), to study and identify the raw material, decoction of Angelica sinensis, and different segmented products of AB-8 macroporous resin. Among them, the coefficient of determination (R^2^) between the infrared spectrum and sucrose was 0.8456 [19]. Craig, AP et al. used MIR to predict the quality of Arabica coffee beans at different roasting degrees. Combined with principal component analysis, Arabica coffee beans and Robusta coffee beans could be distinguished, and high-quality and low-quality coffee beans could also be differentiated [20]. Bureau, S et al. evaluated the possibility of using MIR to analyze the composition of sugars, organic acids, and polyphenols in apples. The regression model showed a good ability to estimate the contents of sugars and organic acids (R^2^ ≥ 0.96) [21]. In addition to using a single instrument, the combined use of MIR and other instruments is also quite widespread. For example, MIR and Raman spectroscopy have good detection and prediction capabilities for the mildewing process of Chinese medicinal herb slices [22]; combining near-infrared and MIR can predict the contents of eight main effective compounds in *Lonicera japonica* and *Artemisia annua* [23]; and combining MIR, ultraviolet, and flow injection analysis can be used to monitor the quality consistency of Chinese herbal medicines in the proprietary Chinese medicine Weibizhi tablets [24].

Based on the aforementioned research on the quality evaluation of TCM, this study focused on the seeds of *Cichorium glandulosum Boiss. et Huet.* as the main research object. Firstly, based on UPLC-MS/MS, combined with database matching, the possible main chemical components were analyzed. Then, the chemical components were confirmed again using HPLC with chemical standards. Five of these components were selected as potential quality markers, and quantitative models for them were established by using MIR and NIR combined with chemometrics, providing a theoretical basis for the rapid quality evaluation of CS.

## 2. Materials and Methods

### 2.1. Reagents and Medicinal Materials

Esculin (110,740–201,806, 92.4%), 4-hydroxybenzoic acid (101,149–202,204, 100%), chlorogenic acid (110,753–202,119, 96.3%), esculetin (110,741–202,109, 98.3%), chicoric acid (111,752–202,105, 98.3%), quercetin (100,081–201,610, 99.3%), isorhamnetin (110,860–201,410, 99.1%), caffeic acid (110,885–200,102, 99.7%), kaempferol (110,861–202,013, 97.4%), apigenin (111,901–201,102, 98.4%), hyperoside (111,521–201,809, 94.7%), and isoquercitrin (111,809–202,205, 96.3%) were purchased from the National Institutes for Food and Drug Control, China. Isochlorogenic acid A (95.0%), isochlorogenic acid B (98.0%), isochlorogenic acid C (98.0%), 1,4-dicaffeoylquinic acid (95.0%), 1,5-dicaffeoylquinic acid (98.0%), lactucopicrin (98.0%), and lactucin (98.0%) were purchased from Chengdu Push Biotechnology Co., Ltd. (Chengdu, China). α-linolenic acid (98.0%) was purchased from Shanghai Source Leaf Biotechnology Co., Ltd. (Shanghai, China). 

Petroleum ether, ethyl acetate, n-butanol, and absolute ethanol were of analytical grade. Methanol, phosphoric acid, and acetic acid were of chromatographic grade, and water was ultra-pure.

Nineteen batches of CS were identified as dried mature CS by researcher He Jiang at the Xinjiang Uighur Autonomous Region Institute of Medicinal Plants. All samples were dried naturally (for around 7 days) in a cool and ventilated place after collection. The CS was then ground into powder by using a grinder and passed through a 40-mesh sieve before being placed in a desiccator at room temperature for later use. Detailed collection information for the 19 batches of CS is provided in Table S1.

### 2.2. Instruments

UltiMate 3000 UHPLC-MS/MS (Thermo Fisher Scientific, Waltham, MA, USA). Agilent 1260 HPLC (Agilent Technologies, Santa Clara, CA, USA). MS105DU electronic balance (Mettler Toledo, Greifensee, Switzerland). FOLI10 Fourier transform infrared spectrometer (Shanghai Yingsha Optics Technology Co., Ltd., Shanghai, China). UPC-1-10T ultra-pure water system (Sichuan Youpu Ultra-Pure Technology Co., Ltd., Sichuan, China). DZTW temperature-controlled electric heating mantle (Beijing Yongguangming Medical Instruments Co., Ltd., Beijing, China). KQ-5200B ultrasonic cleaner (Kunshan Ultrasonic Instruments Co., Ltd., Kunshan, China). EYELAN-1300 rotary evaporator (Shanghai Ailang Instruments Co., Ltd., Shanghai, China). MS105DU electronic balance (Mettler Toledo, Switzerland). FW-80 high-speed universal grinder (Beijing Yongguangming Medical Instruments Co., Ltd., Beijing, China).

### 2.3. Experimental Methods

#### 2.3.1. Sample Preparation

A total of 10.0 g of CS powder was weighed and added into a conical flask, 30 mL of 95% ethanol was added, and the mixture was soaked for 12 h. The mixture was then subjected to reflux extraction for 3 h, followed by 2 h, and another 2 h, respectively. The filtrates were combined, reduced under pressure to concentrate, and 20 mL of water was added to dissolve. The mixture was then extracted sequentially with equal volumes of petroleum ether, ethyl acetate, and n-butanol, each for three times. The extraction liquids were combined, and the four effective fractions (petroleum ether, ethyl acetate, n-butanol, and water) were evaporated to dryness under reduced pressure. Each fraction was resuspended in 5 mL of methanol, mixed in a ratio of 8:1:4:4, and filtered through a 0.45 μm microporous filter membrane to obtain the sample for UHPLC-MS/MS analysis.

A total of 2.0 g of powder was weighed from each of the 19 batches of CS power, 20 mL of 60% ethanol was added, and the mixture was heated under reflux conditions for extraction for 3 h. After cooling to room temperature, the mixture was filtered, and then the pressure was reduced to evaporate the filtrate to dryness. The residue was transferred to a 5 mL volumetric flask, made up to the mark with methanol, mixed thoroughly, and filtered through a 0.45 μm microporous filter membrane. The filtrate was then taken for HPLC analysis.

Esculin, 4-hydroxybenzoic acid, chlorogenic acid, esculetin, caffeic acid, lactucin, cichoric acid, 1,4-dicaffeoylquinic acid, isochlorogenic acid B, isochlorogenic acid A, 1,5-dicaffeoylquinic acid, hyperoside, isoorientin, isochlorogenic acid C, quercetin, lactucopicrin, kaempferol, apigenin, isorhamnetin, and α-linolenic acid was weighed, and a mixed reference solution was prepared with methanol at concentrations of 0.0260, 0.0243, 0.0267, 0.0282, 0.0259, 0.0176, 0.0286, 0.0249, 0.0160, 0.0270, 0.0275, 0.0247, 0.0257, 0.0270, 0.0255, 0.0284, 0.0256, 0.0245, 0.0262, and 0.0250 mg/mL, respectively, for the identification of compounds in the CS.

#### 2.3.2. The Condition of UPLC-MS/MS

Chromatographic column: YMC-PACK ODS-A (250 mm × 4.6 mm, 5 μm). Mobile phase: 0.2% phosphoric acid solution (A)–methanol (B). Gradient elution: 0~12 min, 5~17% (B); 12~20 min, 17~35% (B); 20~40 min, 35~45% (B); 40~50 min, 45~60% (B); 50~60 min, 60~100% (B); 60~70 min, 100% (B). Flow rate: 1.0 mL/min. Detection wavelength: 254 nm. Column temperature: 40 °C. Injection volume: 10.0 μL. Ion source: HESI. Negative ion mode: Sheath gas flow rate 35 psi, auxiliary gas flow rate 10 arb, spray voltage 2.8 kV, ion transfer tube temperature 320 °C, auxiliary gas temperature 310 °C, collision energy (NCE): 20, 40, 60 eV. Detection mode: Full MS/dd-MS2, Resolution: 70,000 and 17,500. Scan range: *m*/*z* 100~1500. Positive ion mode: Sheath gas flow rate 35 psi, auxiliary gas flow rate 10 arb, spray voltage 3.3 kV, ion transfer tube temperature 320 °C, auxiliary gas temperature 310 °C, NCE: 20, 40, 60 eV. Detection mode: Full MS/dd-MS2. Resolution: 70,000 and 17,500. Scan range: *m*/*z* 100~1500. Data acquisition was performed using Thermo Xcalibur software (v4.3, Thermo Fisher Scientific).

The CS samples were analyzed using UHPLC-MS/MS in both positive and negative ion modes. The Thermo Xcalibur software was employed to collect and analyze the original mass spectrometry data, yielding information such as retention times, mass-to-charge ratios, peak intensities, and compound ion peaks. This mass spectrometry analysis enabled the determination of highly reliable chemical components presented in CS.

#### 2.3.3. The Condition of HPLC

The chromatographic separation was performed using a YMC-PACK ODS-A column (250 mm × 4.6 mm, 5 μm) with a mobile phase consisting of 0.2% phosphoric acid solution (A) and methanol (B). A gradient elution program was applied as follows: 0–5 min, 5–15% (B); 5–10 min, 15–20% (B); 10–15 min, 20–27.5% (B); 15–20 min, 27.5–30% (B); 20–30 min, 30–37% (B); 30–40 min, 37% (B); 40–45 min, 37–50% (B); 45–50 min, 50–60% (B); 50–60 min, 60–65% (B); 60–65 min, 65–85% (B); 65–70 min, 85–100% (B); and 70–80 min, 100% (B). The flow rate was maintained at 1.0 mL/min, and the detection wavelength was set at 254 nm. The column temperature was controlled at 40 °C, and the injection volume was 5.0 μL.

#### 2.3.4. Mid-Infrared Spectrometer

The FOLI10 Fourier Transform Infrared Spectrometer (INSA Optics, Qingpu District, Shanghai, China) was preheated for 30 min before use. The CS powder was placed on the sample cell of the diamond single reflection attenuated total reflectance accessory equipped with the spectrometer. The measurement was conducted with a high-sensitivity DLaTGS detector (Bruker Optics, Billerica, MA, USA). The scan range was set from 4000 to 650 cm^−1^, with 32 scans per measurement, and a resolution of 4.0 cm^−1^. A background spectrum was collected every hour, and each sample was collected 45 spectra. The average spectrum was calculated for subsequent modeling and analysis.

#### 2.3.5. Algorithms

Partial Least Squares Regression (PLSR) is a multivariate regression analysis method that constructs a component matrix to perform regression analysis and data compression simultaneously, aiming to solve multivariate linear regression problems. In this research, MIR spectroscopy was used to establish prediction models for five chemical components in CS, with the goal of achieving rapid quality analysis of CS.

Infrared spectra contain a large number of spectral bands, some of which may contribute little to the predictive power of the model and could even introduce noise. By using variable selection algorithms, the bands that make a significant contribution can be identified and selected, thereby improving the predictive performance of the model. In this study, three algorithms with completely different design principles were selected to optimize the performance of the model: (1) Variable selection algorithm for a single interval: Moving Window Partial Least Squares (MWPLS) is an algorithm proposed by the research group of Yuejin Wu, which selects the most relevant variables to the target variable by sliding a fixed window over the spectra. Although MWPLS can accelerate the operation speed of the model and improve its prediction performance to a certain extent, the single region selected may not meet the requirements of both the training set and the test set simultaneously, resulting in over-fitting or under-fitting of the model [25]; (2) Variable selection algorithm for removing irrelevant information: Uninformative Variables Elimination-Successive Projections Algorithm (UVE-SPA) introduces the SPA algorithm based on UVE, which not only reduces the computational complexity during the feature selection process but also improves the robustness of the model, reducing sensitivity to noise and outliers. By removing noise information and collinearity in the vector space to find variable information related to chemical values, the performance of the model is generally excellent, but the running time is usually longer [26]; and (3) Variable selection algorithm for interval combination optimization: The Interval Combination Optimization (ICO) algorithm, proposed by Song et al., considers the impact of different interval combinations on the model results by generating a large number of combinations between intervals. Since the variable subset with the strongest correlation with the training set indicators is selected, there is a high likelihood of excessive data dependence on the training set, which may lead to over-fitting of the model [27]. In this study, MWPLS, UVE-SPA, and ICO algorithms were utilized for variable selection of infrared spectra to improve the predictive performance of the model results.

During the data acquisition process, not only are there signals from the sample itself, but also instrumental noise that may interfere with the sample information. To minimize the interference from irrelevant information, various preprocessing methods are often combined to optimize the raw data, facilitating subsequent data analysis and modeling. Savitzky–Golay smoothing (S-G smoothing) is commonly used as one of the most popular methods for noise reduction [28]. Standard normal variate (SNV) transformation and multivariate scatter correction (MSC) are primarily used to eliminate the effects of solid particle size, surface scattering, and optical path changes on near-infrared diffuse reflectance spectroscopy. Since the samples collected in this study are powders of CS, S-G smoothing, SNV, and MSC were selected for spectral preprocessing, respectively, in this study for subsequent modeling analysis.

#### 2.3.6. Modeling Information

Due to the relatively small amount of data available for modeling, the Kennard–Stone (K-S) sampling method was selected in this study to ensure the effective selection of representative samples [29]. Four out of 19 batches of CS samples were selected as the test set, with the remaining 15 samples serving as the training set. A five-fold cross-validation was employed to optimize the model parameters. In this paper, MATLAB R2023b was employed for preprocessing, variable selection algorithms, and the PLSR model.

The performance of the model is evaluated through the coefficient of determination of the training set (R^2^_cal_), coefficient of determination of the test set (R^2^_PRE_), cross-validation root mean square error (RMSECV), prediction root mean square error (RMSEP), relative performance deviation (RPD), and relative error rate (RER). The closer R^2^ is to 1, and the closer RMSE is to 0, the better the performance of the model. RPD values should be higher than 2.4 to describe appropriate models [30,31], while an RER above 10 is roughly an indicator of a model with good predictive ability [31,32]. The specific formulas are as follows:

$$R^{2}=1-\frac{\sum_{i=1}^{n}{\left(y_{i,actual}-y_{i,predicted}\right)}^{2}}{\sum_{i=1}^{n}{\left(y_{i,actual}-\bar{y_{i,actual}}\right)}^{2}}$$

$$RMSECV=\sqrt{\frac{\sum_{i=1}^{n}{\left(y_{i,actual}-y_{i,predicted}\right)}^{2}}{n-1}}$$

$$RMSEP=\sqrt{\frac{\sum_{i=1}^{m}{\left(y_{i,actual}-y_{i,predicted}\right)}^{2}}{m-1}}$$

$$RPD=\frac{SD}{RMSE}$$

$$SD=\sqrt{\frac{\sum_{i=1}^{n}{\left(y_{i,actual}-\bar{y_{i,actual}}\right)}^{2}}{n-1}}$$

$$RER=\frac{y_{acual,max}-y_{actual,min}}{RMSE}$$

wherein,${\;y}_{i,actual}$ represents the actual value of the $i_{th}\;$ sample. $y_{i,predicted}$ represents the predicted value of the $i_{th}\;$ sample. n and m are the numbers of the training set and test set, respectively. $y_{acual,max}$ and $y_{acual,min}$ refer to maximum and minimum values in the actual values.

## 3. Results

### 3.1. UPLC-MS/MS

**Figure 1:**
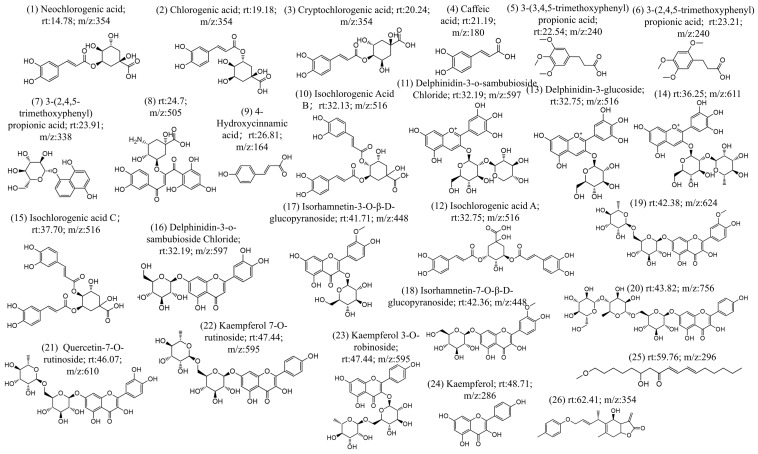
26 compounds were identified based on UPLC-MS/MS analysis.

The chemical constituents of CS were investigated using UHPLC-MS/MS, resulting in ion chromatograms and additional positive (Figure S1) and negative ion mode diagrams (Figure S2). The compound information contained in the MS/MS diagrams was analyzed through two methods. In the first method, the MS/MS diagrams were analyzed manually (first, identifying the parent ion peak through the MS, and then determining the fragment ion peaks using secondary mass spectrometry (MS/MS), thereby deducing the structure of the compound), which confirmed the specific compound structure. The second method was database searching (using Compound Discoverer), which involved matching the MS/MS fragments with a database and determining the compound information based on the level of confidence. Regarding the manual analysis, the MS/MS data were analyzed based on the characteristic ion information of the compounds, resulting in the identification of 26 compound structures (Figure 1). The process of the analysis is shown in Figure S1. Compared with the compound database, 20 of the compounds were found to be already present and retrievable (Figure 1). In the database comparison method, retention time, mass-to-charge ratio, ion peaks, and other information were used for database comparison. The results were organized in combination with literature and comparison confidence levels. By integrating the results of manual analysis and database comparison, 49 chemical components were obtained, as shown in Table 1. In order of retention time, these includ isoguanosine, neochlorogenic acid, p-hydroxybenzoicacid, esculin, l-hyoscyamine, caffeoylquinicacid, esculetin, cryptochlorogenicacid, caffeicacid, 1,3-dicaffeoylquinicacid, 3-(3,4,5-trimethoxyphenyl)propanoic acid, 3-(3,4,5-trimethoxyphenyl)propanoic acid, lactucin, hydrojuglone glucoside, p-hydroxy-cinnamicacid, chicoric acid, isochlorogenicacid b, delphinidin3-o-beta-d-sambubioside, isochlorogenicacid a, 1,5-dicaffeoylquinicacid, delphinidin3-glucoside, quercetin-3-o-glucoside, quercetin-3-o-galactoside, rutin, isochlorogenicacid c, trifolin, luteolin-4′-o-glucoside, morin, kaempferol-7-o-hexoside, moupinamide, luteolin 7-glucoside, isorhamnetin-3-o-glucoside, kaempferol-3-o-robinobioside, isorhamnetin7-glucoside, ethylcaffeate, 11β,13-dihydrolactucopicrin, quercetin-7-o-rutinoside, quercetin, kaempferol 7-o-rutinoside, lactupicrin, biorobin, kaempferol, d-(-)-salicin, isorhamnetin, 9,12,13-trihydroxy-10-octadecenoicacid, α-linolenicacid, apigenin, linolenicacid ethylester, and 2-linoleoyl glycerol.

In this study, 49 compounds of CS were identified through UHPLC-MS/MS, among which 33 components have not been reported previously. Newly identified compounds include p-hydroxycinnamic acid and salicin, quercetin, kaempferol, isorhamnetin and their corresponding glycosides, lactucin, 11β-13-dihydrolactucin, 1,5-dicaffeoylquinic acid, and cryptochlorogenic acid. Upon reviewing the literature, it was found that comprehensive research reports on the chemical composition of CS are relatively scarce. However, UHPLC-MS/MS could provide insights for the identification of the components of CS.

### 3.2. HPLC for Compound Identification

**Figure 2:**
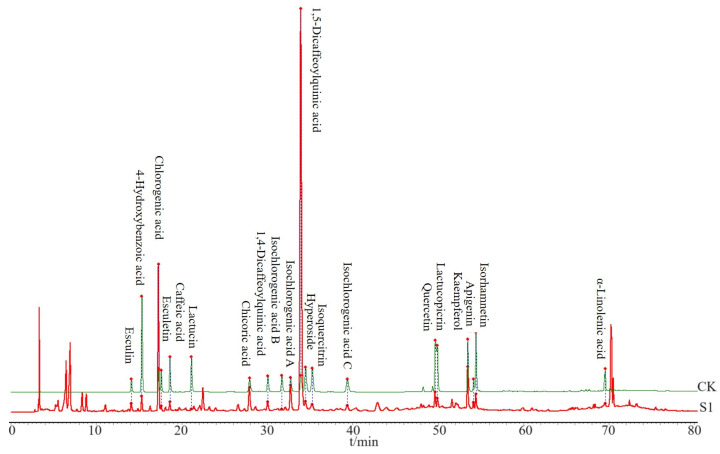
Comparison of HPLC spectra between the CS sample and the standard sample. S1 refers to the first batch of CS, and CK is composed of a mixture of standardized compounds.

The CS sample was prepared according to Section 2.3.1 for HPLC analysis and was analyzed under the chromatographic conditions described in Section 2.3.3. Based on the HPLC obtained from the CS, 20 peaks with distinct peak shapes were selected for analysis. Standard substances were utilized to confirm the chemical compositions present. CK in Figure 2 represents a sample prepared by mixing standard substances (described in Section 2.3.1), and then 20 components in CS (S1) sample were identified (Figure 2), including esculin, 4-hydroxybenzoic acid, chlorogenic acid, esculetin, caffeic acid, lactucin, cichoric acid, 1,4-dicaffeoylquinic acid, isochlorogenic acid B, isochlorogenic acid A, 1,5-dicaffeoylquinic acid, hyperoside, isoorientin, isochlorogenic acid C, quercetin, lactucopicrin, kaempferol, apigenin, isorhamnetin, and α-linolenic acid.

**Figure 3:**
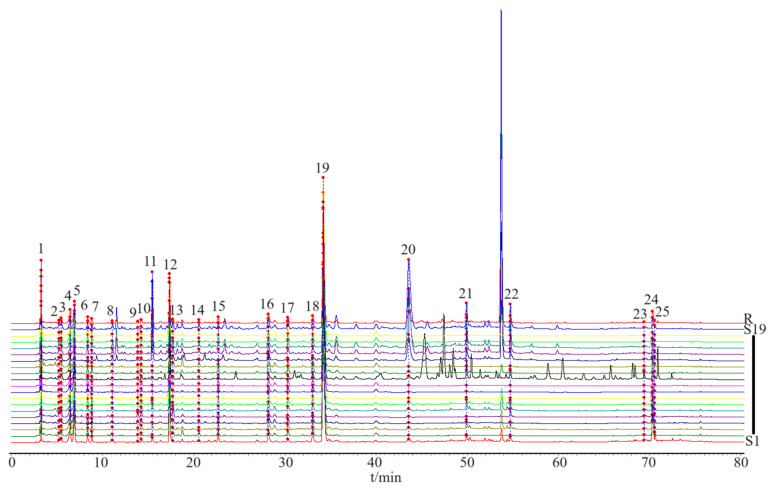
The HPLC fingerprint profiles of 19 batches of CS. S1–S19 refers to the batch of CS, and R is composed of a mixture of standardized compounds.

Based on the determination of the chemical components contained in the HPLC analysis, fingerprint chromatograms were collected for 19 batches of CS (Figure 3). The fingerprint chromatogram-related precision, stability, repeatability, and sample recovery experiments are displayed in the Supplementary Materials Section. Five components with sharp peak shapes and substantial peak areas were selected (chlorogenic acid (peak 12), esculetin (peak 13), 1,4-dicaffeoylquinic acid (peak 17), isochlorogenic acid A (peak 18), and 1,5-dicaffeoylquinic acid (peak 19)) to establish a content determination method and measure their contents in 19 batches of CS.

Chlorogenic acid, esculetin, 1,4-dicaffeoylquinic acid, isochlorogenic acid A, and 1,5-dicaffeoylquinic acid standards were weighed and dissolved in methanol to prepare mixed standard solutions, with concentrations of 0.534 mg/mL for chlorogenic acid, 0.141 mg/mL for esculetin, 0.1245 mg/mL for 1,4-dicaffeoylquinic acid, 0.135 mg/mL for isochlorogenic acid A, and 1.71 mg/mL for 1,5-dicaffeoylquinic acid. The mixed standard solution was diluted with methanol to prepare a series of concentrations. The HPLC conditions were described in Section 2.3.3. The linear regression equations were calculated with the concentration as the abscissa (X) and the peak area as the ordinate (Y). The results are shown in Table S2. The R^2^ values of the five-component linear equations were all above 0.999, indicating that the equations could be used to accurately calculate the content of the corresponding compounds in different CS batches. Based on this, the powder of 19 batches of CS was precisely weighed and prepared into test solutions according to the method described in Section 2.3.1. The solutions were injected for determination under the chromatographic conditions described in Section 2.3.3.

The peak areas of the five components were measured, and the mass fractions of the five components in each sample were calculated based on the linear regression equations. The results are shown in Table S3. The content ranges for chlorogenic acid were 0.4902~1.1943 mg/g, for esculetin 0.0348~0.2410 mg/g, for 1,4-dicaffeoylquinic acid 0.0540~0.2364 mg/g, for isochlorogenic acid A 0.1047~0.2773 mg/g, and for 1,5-dicaffeoylquinic acid 1.4087~4.3194 mg/g. The wide distribution range of the five components is highly representative and meets the requirements for the sample content distribution in the quantitative model.

Based on the research results obtained by UPLC-MS/MS, HPLC was utilized to conduct measurable verification of the components in CS. By comparing with standard samples, the chemical components corresponding to the peaks in HPLC were identified. Fingerprint profiles for 19 batches of CS were established, and five chemical components were screened based on peak shapes. Standard curves for their peak areas versus substance contents were developed to determine the contents of five components, namely chlorogenic acid, esculetin, 1,4-dicaffeoylquinic acid, isochlorogenic acid A, and 1,5-dicaffeoylquinic acid, in the 19 batches of CS.

### 3.3. Infrared Spectroscopy

The 19 batches of CS were divided into a training set and a test set according to the K-S sampling. The content ranges for the 15 training set samples were as follows: chlorogenic acid 0.4902~1.1943 mg/g, esculetin 0.0348~0.2410 mg/g, 1,4-dicaffeoylquinic acid 0.0540~0.2364 mg/g, isochlorogenic acid A 0.1047~0.2773 mg/g, and 1,5-dicaffeoylquinic acid 1.4087~4.3194 mg/g. For the four test set samples, the content ranges were: chlorogenic acid 0.6124~1.1154 mg/g, esculetin 0.0434~0.1596 mg/g, 1,4-dicaffeoylquinic acid 0.0661~0.1769 mg/g, isochlorogenic acid A 0.1548~0.2372 mg/g, and 1,5-dicaffeoylquinic acid 2.5094~4.1686 mg/g. The distribution of specific chemical values within the dataset is shown in Table S4.

#### 3.3.1. Mid-Infrared Spectroscopy

**Figure 4:**
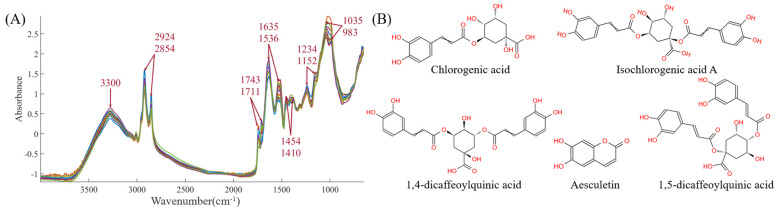
(**A**) The mid-infrared spectra. (**B**) The structures of five chemical components.

The mid-infrared spectra for the 19 batches are shown in the Figure 4A. The functional group information of CS is very rich, mainly concentrated at 3300, 2924, 2854, 1743, 1711, 1635, 1536, 1454, 1410, 1234, 1152, 1035, and 983 cm^−1^, corresponding to -O-H, -C-H, -C=O, -C=C, -CH_2_, -CH_3_, -C-O, -C-N, and other functional groups, respectively. These spectra directly or indirectly reflect the chemical bond information of the five indicators: chlorogenic acid, esculetin, 1,4-dicaffeoylquinic acid, isochlorogenic acid A, and 1,5-dicaffeoylquinic acid. The detailed chemical structures of the five components are provided in Figure 4B. Therefore, subsequent efforts will involve using chemometric methods (such as preprocessing, variable selection, and multivariate analysis algorithms) to establish prediction models between infrared spectra and the five chemical components, with the aim of accelerating the rapid detection and quality evaluation of CS chemical components.

#### 3.3.2. Infrared Models for Chemical Components in CS

- Chlorogenic Acid

**Figure 5:**
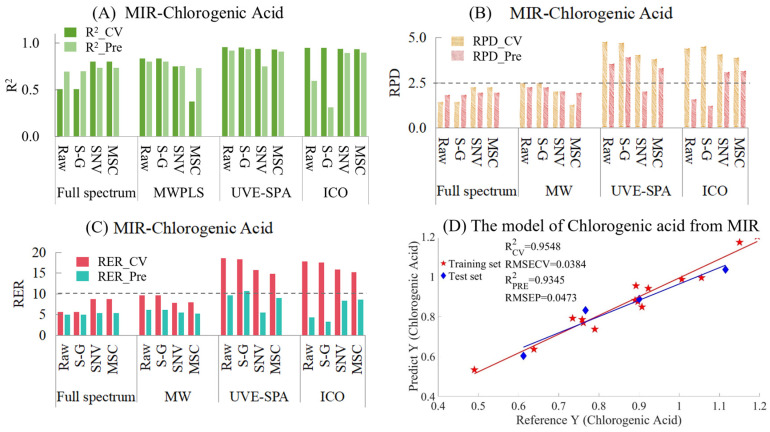
Mid-infrared model results of chlorogenic acid. (**A**) R^2^ result. (**B**) RPD results. (**C**) RER results. (**D**) Optimal model results.

The results of MIR models for chlorogenic acid are provided in Table S5 and Figure 5. Figure 5A shows the model results of different preprocessing methods combined with different variable selection algorithms. Among them, for the full-spectrum data, the performance of the model after SNV and MSC preprocessing was better than that of the raw and S-G smoothed data. However, MWPLS gave the opposite result, where the original and smoothed data performed better than the data preprocessed by SNV and MSC. This might be because MWPLS only selected one wavelength interval, while SNV and MSC were global transformations based on the entire spectral data. This might have led to the situation in the training set where the window selected by MWPLS was only suitable for some samples and could not meet the requirements of both the training set and the test set simultaneously. When UVE-SPA was combined with the three preprocessing methods, the overall performance of the model was excellent. As for the ICO algorithm, there was an overfitting situation for both the raw and smoothed data, while the performances of MSC and SNV were relatively excellent. When comparing the RPD values of the models (Figure 5B), the RPD values of UVE-SPA combined with the original data and S-G smoothing, as well as ICO combined with SNV and MSC, were all greater than 2.5, indicating that the models had good applicability. When comparing the RER values of the models (Figure 5C), since the differences between the maximum and minimum values of the training set and the test set were 0.7041 and 0.5030, respectively, the overall RER of the training set was larger than that of the test set. Among them, only the RER value of the test set of UVE-SPA combined with S-G smoothing was higher than 10, which indicated that the model had good predictive ability. The corresponding R^2^ values of the training set and the test set were 0.9548 and 0.9345, respectively. The above model results indicate that there are significant differences in the model results when different variable selection algorithms were combined with different preprocessing methods. For the chlorogenic acid index, UVE-SPA combined with S-G smoothing could obtain relatively excellent model results. The R^2^ values of both the training set and the test set were above 0.93, the RPD values were all greater than 2.5, and the RER values were all greater than 10.

- Aesculin

**Figure 6:**
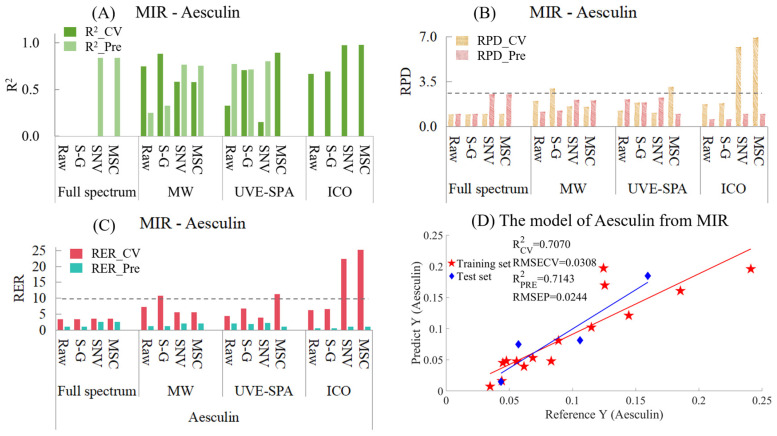
Mid-infrared model results of Aesculin. (**A**) R^2^ result. (**B**) RPD result. (**C**) RER results. (**D**) Optimal model results.

Similarly, the MIR models for aesculin are provided in Table S6 and Figure 6. Among the models with three variable selection methods combined with three preprocessing methods (Figure 6A), the performances of many models were very poor, and the R^2^ values were negative. Therefore, at that time, the models with negative R^2^ values were no longer presented. It can be seen that for the full-spectrum data, the models of the raw data and the data after S-G smoothing had negative R^2^ values for both the training set and the test set. However, for SNV and MSC, the models showed strong under-fitting, which might have been caused by the relatively small data volume of the test set and the chemical values of some samples in the test set being quite close to those in the training set. As for the four models of ICO, all of them showed the situation of over-fitting. This might have been because ICO used the data from the training set to find the variable points that had the best fitting effect with the chemical values, which might have led to an overly close fitting and caused these variable points to have poor prediction on unknown samples. In the MWPLS model, overfitting occurred with the raw data and the data after S-G smoothing, while SNV and MSC showed a certain degree of under-fitting. The results suggested that a single spectral region may be insufficient for accurate prediction of the Aesculin index. Among all datasets analyzed by UVE-SPA, the S-G smoothed data yielded the most optimal modeling performance. There was neither overfitting nor underfitting, and the R^2^ values of the training set and the test set were 0.7070 and 0.7143, respectively (Figure 6D). In terms of the RPD (Figure 6B), among the constructed models, the RPD values of neither the training set nor the test set of any model exceeded 2.5. The RPD value of the model with UVE-SPA combined with the S-G model was around 1.8, which indicated that there was a certain correlation between the observed values and the predicted values. However, the applicability of the model was not excellent, and other methods were needed to optimize the model. Regarding the RER (Figure 6C), the differences between the maximum and minimum values of the training set and the test set were 0.2062 and 0.1162, respectively. The RER values of the test sets of all models were less than 10, and the optimal RER values were only concentrated around 2.5. This indicates that the model for establishing the RER using MIR needs to be optimized.

- 1,4-Dicaffeoylquinic Acid

**Figure 7:**
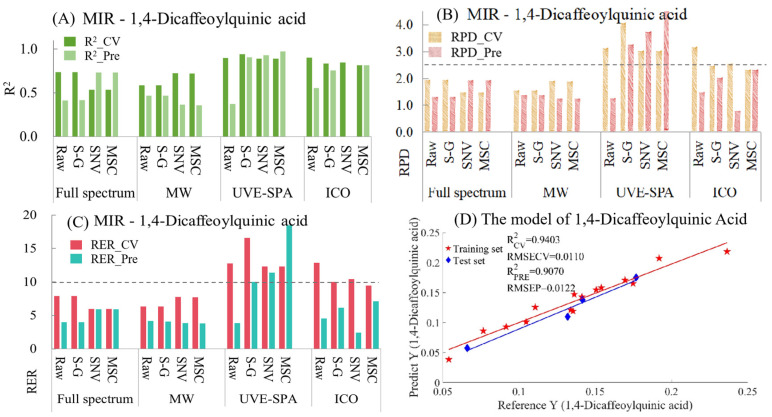
Mid-infrared model results of 1,4-dicaffeoylquinic acid. (**A**) R^2^ result. (**B**) RPD result. (**C**) RER results. (**D**) Optimal model results.

For 1,4-dicaffeoylquinic acid, the model indicators of MIR are summarized in Table S7 and Figure 7. From Figure 7A, in the models of full-spectrum data, the raw data and the data after S-G smoothing showed overfitting, while the data processed by SNV and MSC presented underfitting. Compared with the models of full-spectrum data, the performance of the MWPLS models of the original data and the data after S-G smoothing was better (manifested as a decrease in the degree of overfitting and an increase in the R^2^ value of the test set). For UVE-SPA, except for the raw data, which showed overfitting, the models using S-G smoothing, SNV, and MSC performed relatively well. After using the ICO algorithm, the data preprocessed by SNV had obvious overfitting. Compared with the models of the original data, the performance of the models using S-G smoothing and MSC was better. Regarding the RPD (Figure 7B) and RER values (Figure 7C), only when UVE-SPA was combined with S-G smoothing, SNV, and MSC, the RPD values of both the training set and the test set were greater than 2.5, and the RER values were greater than 10. This indicates that, for 1,4-dicaffeoylquinic acid, the model has good applicability and excellent prediction ability. Among the three models that met the requirements, the model result of UVE-SPA combined with S-G smoothing was selected for display (Figure 7D). The R^2^ values for the training set and the test set were 0.9403 and 0.9070, respectively, indicating a good linear relationship between the predicted values and the true values.

- Isochlorogenic Acid A

**Figure 8:**
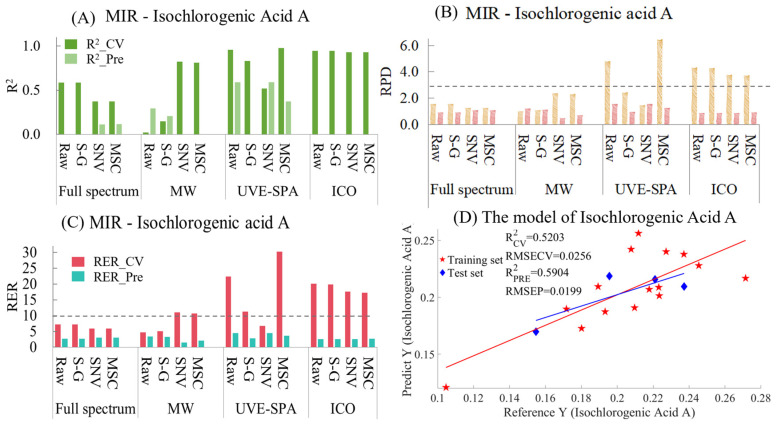
Mid-infrared model results of Isochlorogenic Acid A. (**A**) R^2^ result. (**B**) RPD result. (**C**) RER results. (**D**) Optimal model results.

The MIR model indicators for isochlorogenic acid A are summarized in Table S8 and Figure 8. As shown in Figure 8A, in the full-spectrum data, the original data and the data after S-G smoothing showed obvious overfitting, and the R^2^ values of the test set were all negative. Although the R^2^ values of the test set were positive after processing with SNV and MSC, they were only around 0.1, and the models hardly had any predictive ability. In MWPLS, the data processed by SNV and MSC showed overfitting, with the R^2^ values of the test set being negative. The original data presented underfitting, and for the data after S-G smoothing, the model performance was poor, with the R^2^ values of both the training set and the test set being less than 0.25. In UVE-SPA, the raw data, the data after S-G smoothing, and the MSC-processed data showed overfitting. Only the SNV model performed normally, but the R^2^ values of the training set and the test set were only 0.5203 and 0.5904, respectively (Figure 8D). ICO showed obvious overfitting. Regarding the essence of its algorithm, it generated countless variable combination subsets and found the subset that had the best fitting effect with the training set. However, its prediction performance for unknown samples was poor, which might have been the reason for the overfitting of the model. As for the RPD values (Figure 8B) and RER values (Figure 8C), the RPD values of the test sets of all models were less than 2.5, and the RER values were all less than 10. This indicates that the existing dataset and modeling strategy can not achieve the prediction of isochlorogenic acid A in CS.

It is worth noting that the chemical structure of chlorogenic acid is very similar to that of isochlorogenic acid A. However, the constructed infrared spectroscopy models differed significantly. The analysis suggested that the reason might be due to the chemical indicators of the 19 batches of isochlorogenic acid A being relatively close, within a small chemical value ranging from 0.1047 to 0.2773. The insufficient chemical range led to poor data model results. In the future, adding more representative samples could help increase the predictive power of the model.

- 1,5-Dicaffeoylquinic acid

**Figure 9:**
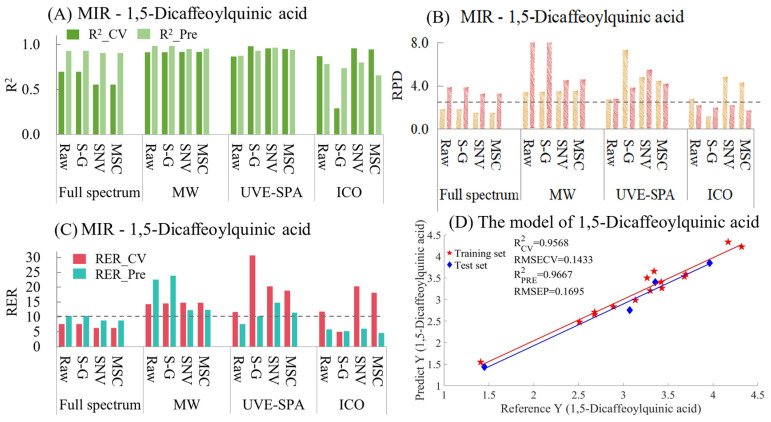
Mid-infrared model results of 1,5-Dicaffeoylquinic acid. (**A**) R^2^ result. (**B**) RPD result. (**C**) RER results. (**D**) Optimal model results.

The MIR model indicators for 1,5-dicaffeoylquinic acid are summarized in Table S9 and Figure 9. As shown in Figure 9A, for the full-spectrum data, the raw data and the data after the three preprocessing methods all showed underfitting. This may have been because the data of the test set were quite close to those of the training set, resulting in the model’s prediction effect being rather biased towards overestimating the performance. Among the four sets of data in MWPLS and UVE-SPA, the overall model performance was relatively excellent. Except for the original data in UVE-SPA, the R^2^ values of the training sets and test sets for the rest were all above 0.9. In the models after ICO, only the model using the original data performed relatively well. The models using S-G smoothing, SNV, and MSC all showed certain degrees of overfitting and underfitting. Regarding the RPD (Figure 9B), the RPD values of the four sets of data in MWPLS and UVE-SPA were all greater than 2.5, which indicated that the models had good applicability. In terms of RER (Figure 9C), in the training set and the test set of 1,5-Dicaffeoylquinic acid, the differences between the maximum and minimum values were 2.9107 and 2.5109, respectively. In the four datasets of MWPLS, the RER values of both the training set and the test set were greater than 10. In UVE-SPA, the RER values of the data of S-G smoothing, SNV, and MSC were also all greater than 10, which indicated that the models had good predictive ability. The optimal model was UVE-SPA combined with the SNV data, and the R2 values of the training set and the test set were 0.9568 and 0.9661, respectively (Figure 9D). The above model results indicate that mid-infrared combined with the UVE-SPA algorithm can be used for the prediction of 1,5-dicaffeoylquinic acid in CS.

Through the combination of mid-infrared spectroscopy with three variable selection methods, namely MWPLS, UVE-SPA, and ICO, and three preprocessing methods, namely S-G smoothing, SNV, and MSC, mathematical fitting models were established for five indexes, including Chlorogenic Acid, Aesculin, 1,4-Dicaffeoylquinic Acid, Isochlorogenic Acid A, and 1,5-Dicaffeoylquinic Acid in CS. Among them, the optimal models for each index are summarized in Table 2. For the three indexes of Chlorogenic Acid, 1,4-Dicaffeoylquinic Acid, and 1,5-Dicaffeoylquinic Acid, the R2 values for both the training set and the test set were above 0.9, the RPD values were all greater than 2.5, and the RER values were greater than 10. This indicates that the combination of mid-infrared spectroscopy and chemometrics has excellent model applicability and prediction performance for these three indexes, and can provide technical support for the rapid detection of Chlorogenic Acid, 1,4-Dicaffeoylquinic Acid, and 1,5-Dicaffeoylquinic Acid in CS in follow-up research. However, for Aesculin and Isochlorogenic Acid A, the R^2^ values for both the training set and the test set were above 0.8, but the RPD values were all less than 2, and the RER values were less than 7. This indicates that the accurate prediction of these two components could not be achieved using the existing instruments and data analysis methods. Analyzing the reasons, for Aesculin, it might have been because among the 19 CS samples, its chemical values were distributed in the range of 0.05–0.25, and the chemical values of a large number of samples were concentrated in the range of 0.05–0.1. On the one hand, the content of the chemical components was relatively low. On the other hand, the chemical values of the samples were rather concentrated and not representative, which led to the poor performance of the model. For follow-up research on Aesculin, the number of CS samples could be increased to make the distribution range of its chemical values wider, thus improving the model fitting performance. As for Isochlorogenic Acid A, there was also the problem of weak representativeness of the samples. The distribution range of the chemical values of the samples was from 0.1 to 0.28, and the vast majority of the samples were distributed between 0.15 and 0.25. This made the chemical values of the samples concentrated, and the model was unable to fully learn the internal laws of the data, resulting in poor model fitting.

When comparing the three variable selection algorithms, the model performance obtained by the UVE-SPA algorithm was the most excellent. In terms of the algorithm design principle, UVE-SPA first used UVE to eliminate the variable points whose information contribution was less than that of the noise. Then, it utilized the SPA algorithm to minimize the collinearity in the vector space and select the variables that were most representative of the target variable. However, the model always showed the situation of overfitting when using the ICO algorithm. This might have been because ICO generated several variable subsets and searched for the subset that was most relevant to the chemical values of the training set. As a result, the model relied too much on the samples in the training set and had a poor fitting degree for unknown samples, thus exhibiting the overfitting phenomenon. As for MWPLS, the model often showed the situations of overfitting or underfitting. It divided the spectrum into several intervals and only used one of the intervals to establish the model. This might have led to the selected interval being only applicable to the training set or the test set, lacking comprehensive consideration.

## 4. Conclusions

This study focused on the Uyghur herbal medicine *C. glandulosum* seed as the main research object. Utilizing UPLC-MS/MS, a compositional analysis was conducted. Through mass spectrometry interpretation and database comparison, 20 important chemical components were preliminarily identified. Subsequently, HPLC was employed to confirm the identified components with reference standards, establishing a fingerprint profile for 19 batches of samples and determining the content of five components: chlorogenic acid, esculetin, 1,4-dicaffeoylquinic acid, isochlorogenic acid A, and 1,5-dicaffeoylquinic acid. Using the abundant components measured by HPLC as indicators, MIR was combined with three variable selection algorithms and three preprocessing methods to build predictive models. Among these, for the three indexes of Chlorogenic Acid, 1,4-Dicaffeoylquinic Acid, and 1,5-Dicaffeoylquinic Acid, the R2 values of both the training set and the test set were above 0.9, the RPD values were all greater than 2.5, and the RER values were greater than 10. This indicates that the combination of mid-infrared spectroscopy and chemometrics provides excellent model applicability and prediction performance for these three indexes, offering technical support for the rapid detection of Chlorogenic Acid, 1,4-Dicaffeoylquinic Acid, and 1,5-Dicaffeoylquinic Acid in CS for follow-up research.

In summary, following the principles of “traceability”, “measurability”, and “effectiveness” in quality marker research, this study identified quality markers and established a stable, reliable, and rapid quality evaluation method for Cichorium intybus seed based on these quality markers, providing a reference for the quality control research of CS. However, many ion peaks in UPLC-MS/MS had not yet been structurally and chemically identified, warranting more detailed research. In the data modeling of the five chemical components using infrared spectroscopy, the number of samples was limited, which may have contributed to poor predictive performance for isochlorogenic acid A and Aesculin. More representative samples need to be collected and added to the model in future studies to enhance the stability and predictive performance of the model.

## Figures and Tables

**Table 1:** High-confidence chemical components of CS.

Number	Name	Retention Time (min)	*m*/*z*	Ion Mode	Number	Name	Retention Time (min)	*m*/*z*	Ion Mode
1	Isoguanosine	7.76	284.1	M+H	26	Trifolin	38.01	447.09	M-H
2	Neochlorogenic acid	14.78	355.31	M+H	27	Luteolin-4′-O-glucoside	39.91	449.11	M+H
3	p-Hydroxybenzoic acid	16.07	137.02	M-H	28	Morin	40.31	301.05	M-H
4	Esculin	16.54	339.07	M-H	29	Kaempferol-7-O-hexoside	40.71	449.11	M+H
5	L-Hyoscyamine	17.79	290.17	M+H	30	Moupinamide	41.39	314.14	M+H
6	Caffeoylquinic acid	19.18	355.1	M+H	31	Luteolin 7-Glucoside	41.52	449.1	M+H
7	Esculetin	20.22	178.03	M+H	32	Isorhamnetin-3-O-Glucoside	41.71	477.1	M-H
8	Cryptochlorogenic acid	20.24	355.31	M+H	33	Kaempferol-3-O-Robinobioside	41.81	595.17	M+H
9	Caffeicacid	21.19	181.05	M+H	34	Isorhamnetin7-Glucoside	42.36	479.11	M+H
10	1,3-Dicaffeoylquinic acid	21.69	517.13	M+H	35	Ethylcaffeate	45.01	207.07	M-H
11	3-(3,4,5-Trimethoxyphenyl) propanoic acid	22.54	241.25	M+H	36	11beta,13-Dihydrolactucopicrin	46	413.4	M+H
12	3-(3,4,5-Trimethoxyphenyl) propanoic acid	23.21	241.25	M+H	37	Quercetin-7-O-rutinoside	46.07	611.15	M+H
13	Lactucin	23.83	277.11	M+H	38	Quercetin	46.31	303.05	M+H
14	Hydrojuglone glucoside	23.91	163.04	M-H	39	Kaempferol 7-O-Rutinoside	47.44	595.14	M+H
15	p-Hydroxy-cinnamicacid	26.81	163.04	M-H	40	Lactupicrin	48.17	411.24	M+H
16	Chicoric acid	28.30	473.07	M-H	41	Biorobin	48.38	595.15	M+H
17	Isochlorogenicacid B	32.13	517.45	M+H	42	Kaempferol	48.71	285.04	M-H
18	Delphinidin3-O-beta-D-sambubioside	32.19	597.15	M	43	D-(-)-salicin	48.83	309.1	M+Na
19	IsochlorogenicacidA	32.75	517.45	M+H	44	Isorhamnetin	49.28	315.05	M-H
20	1,5-Dicaffeoylquinic acid	33.69	517.13	M+H	45	9,12,13-Trihydroxy-10-OctadecenoicAcid	54.71	329.23	M-H
21	Delphinidin3-glucoside	35.63	465.11	M	46	Alpha-Linolenicacid	57.89	279.23	M+H
22	Quercetin-3-O-glucoside	35.64	465.1	M+H	47	Apigenin	59.36	269.05	M-H
23	Quercetin-3-O-galactoside	35.66	463.09	M-H	48	Linolenicacidethylester	61.68	307.26	M+H
24	Rutin	36.22	609.15	M-H	49	2-LinoleoylGlycerol	62.72	355.28	M+H
25	Isochlorogenic acidC	37.76	517.45	M+H					

**Table 2:** Summary table of MIR optimal model results for five indicators.

Components	R^2^cv	RMSECV	RPD_cv	RER_cv	R^2^pre	RMSEP	RPD_pre	RER_pre
Chlorogenic Acid	0.9548	0.0384	4.7036	18.3359	0.9345	0.0473	3.9073	10.6342
Aesculin	0.7070	0.0308	1.8474	6.6948	0.7143	0.0244	1.8709	4.7623
1,4-Dicaffeoylquinic Acid	0.9403	0.0110	4.0927	16.5818	0.9070	0.0122	3.2791	10.0082
Isochlorogenic Acid A	0.5203	0.0256	1.4438	6.7422	0.5904	0.0199	1.5625	4.5226
1,5-Dicaffeoylquinic acid	0.9568	0.1433	4.8113	20.3119	0.9667	0.1695	5.4800	14.8136

## Data Availability

The original contributions presented in this study are included in the article/Supplementary Materials. Further inquiries can be directed to the corresponding authors.

## Supplementary Materials

The following supporting information can be downloaded at: https://www.mdpi.com/article/10.3390/foods14081434/s1, Table S1. Sample information of CS from different batches; Table S2. The results of the linear relationship for the five chemical components; Table S3. Determination results of 5 chemical components in 19 batches of CS samples; Table S4. Distribution of Mid infrared spectral training and test sets in CS samples; Table S5. Infrared model results of chlorogenic acid; Table S6. Infrared model results of Aesculin; Table S7. Infrared model results of 1,4-Dicaffeoylquinic acid; Table S8. Infrared model results of Isochlorogenic acid A; Table S9. Infrared model results of 1,5-Dicaffeoylquinic acid; Figure S1. Positive ion mode spectrum of UPLC-MS/MS; Figure S2. Negative ion mode spectrum of UPLC-MS/MS; Figure S3. The process of mass spectrometry analysis.
