# Supplementary material for: Research on Quality Evaluation of the Seeds of Cichorium glandulosum Boiss. et Huet
Source: Foods. 2025 Apr 21;14(8):1434. doi: 10.3390/foods14081434 (PMC12026558; doi:10.3390/foods14081434)
Supplement: Supplementary file 1 [file foods-14-01434-s001.zip › foods-3540571-supplementary.pdf]

Research on Quality Evaluation of the seeds of *Cichorium glandulosum* *Boiss. et Huet.*

Xu Chen[1,2#], Jianshuang Jiang[3#], Fengling Li[2], Wen Lei[2], Juan Li[2], Juan Li[2], Xiaoting Wang[2], Ayiben·Wenhua[2], Jingjing Xia[2*], Jiang He[1*]

[1]*Key Laboratory of Uygur Medicine, Xinjiang Institute of Materia Medica, No. 140, Xinhua North Road, Tianshan District, Urumqi 830004, China.*

[2]*Xinjiang Key Laboratory of Biological Resources and Genetic Engineering, College of Life Science and Technology & School of Pharmaceutical Sciences and Institute of Materia Medica, Xinjiang University, Urumqi 830046, China.*

[3]*State Key Laboratory of Bioactive Substance and Function of Natural Medicines, Institute of Materia Medica, Peking Union Medical College, Chinese Academy of Medical Sciences, Beijing 100050, China.*

(1) Precision Experiment

The powder of CS (S1) was precisely weighed and prepared into solution according to the method described in section 2.3.1. The solution was injected continuously for six times under the chromatographic conditions described in section 2.3.3. The peak areas of each component were recorded, and the relative standard deviation (RSD) values of the peak areas were calculated. All RSD values were less than 1.36%, indicating that the instrument has good precision.

(2) Stability Experiment

The powder of CS (S1) was precisely weighed and prepared into the solution according to the method described in section 2.3.1. The solution was injected for determination at 0, 2, 4, 8, 12, and 24 hours under the chromatographic conditions described in section 2.3.3. The peak areas of each component were recorded, and the RSD values of the peak areas were calculated. All RSD values were less than 3.57%, indicating that the test solution is stable within 24 hours.

(3) Repeatability Experiment

Six portions of the CS (S1) were precisely weighed and prepared into solutions according to the method described in section 2.3.1. The solutions were injected for determination under the chromatographic conditions described in section 2.3.3. The peak areas of each component were recorded, and the RSD values of the peak areas were calculated. All RSD values were less than 2.16%, indicating that the method has good repeatability.

(4) Sample Recovery Experiment

One gram of the CS powder (S1) was precisely weighed six times, and a certain amount of reference substance was added to each portion. The test solutions were prepared according to the method

described in section 2.3.1 and injected for determination under the chromatographic conditions described in section 2.3.3. The peak areas were recorded, and the recovery rates and RSD values were calculated. The average recovery rates for chlorogenic acid, esculetin, 1,4-dicaffeoylquinic acid, isochlorogenic acid A, and 1,5-dicaffeoylquinic acid were 101.03%, 105.48%, 111.35%, 103.00%, and 103.25%, respectively, with RSD values all less than 5%.

Table.S1 Sample information of *CS* from different batches

Number	Origin	Number	Origin
S1	Jimusar, Changji, Xinjiang Uygur Autonomous Region	S11	Xinjiang Uygur Autonomous Region
S2	Xinjiang Uygur Autonomous Region	S12	Jimusar, Changji, Xinjiang Uygur Autonomous Region
S3	Hetian, Xinjiang Uygur Autonomous Region	S13	Hetian, Xinjiang Uygur Autonomous Region
S4	Hetian, Xinjiang Uygur Autonomous Region	S14	Moyu, Hetian, Xinjiang Uygur Autonomous Region
S5	Hetian, Xinjiang Uygur Autonomous Region	S15	Chengdu, Sichuan
S6	Xinjiang Uygur Autonomous Region	S16	Chengdu, Sichuan
S7	Xinjiang Uygur Autonomous Region	S17	Liaoning
S8	Xinjiang Uygur Autonomous Region	S18	Liaoning
S9	Xinjiang Uygur Autonomous Region	S19	Pakistan
S10	Xinjiang Uygur Autonomous Region		

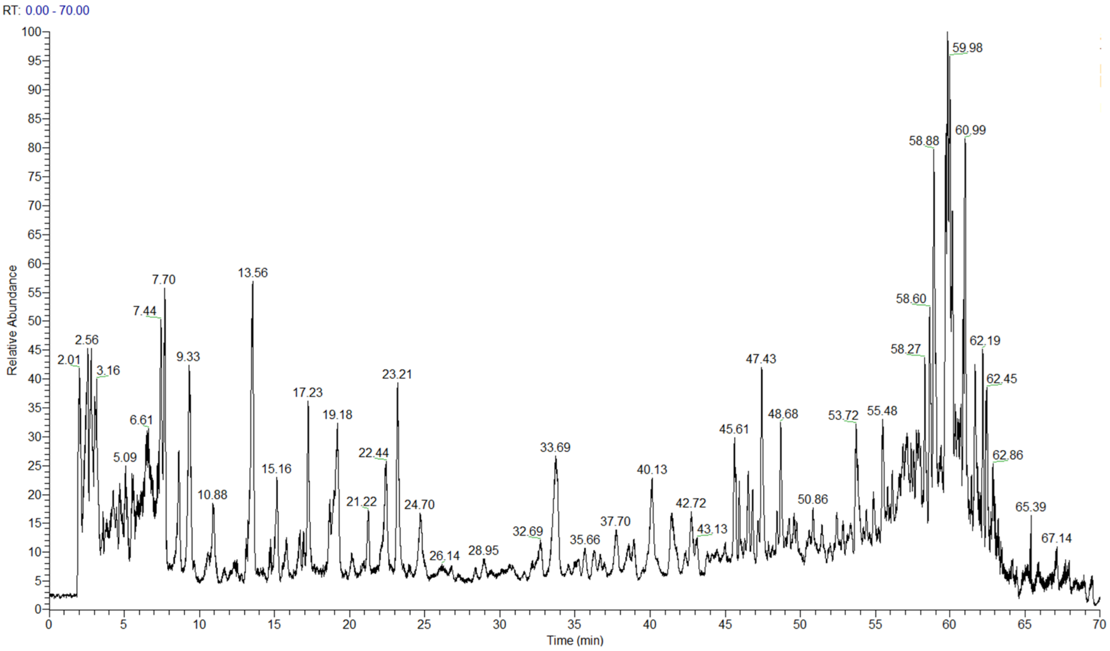

Figure.S1 Positive ion mode spectrum of UPLC-MS/MS.

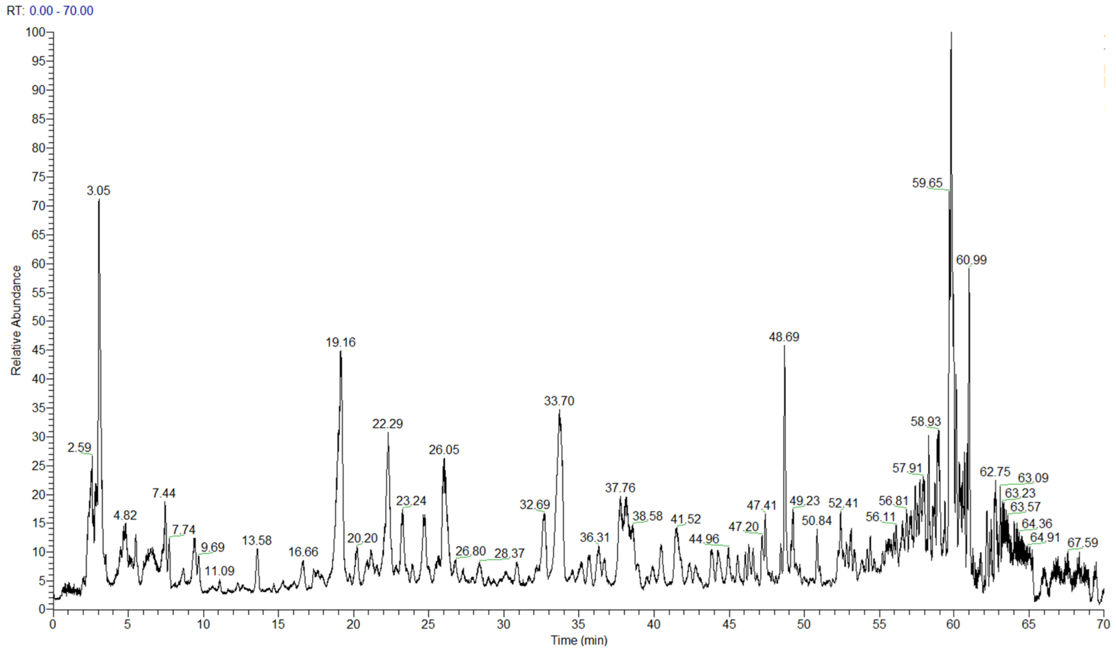

Figure.S2 Negative ion mode spectrum of UPLC-MS/MS.

Fig.S3 The process of mass spectrometry analysis

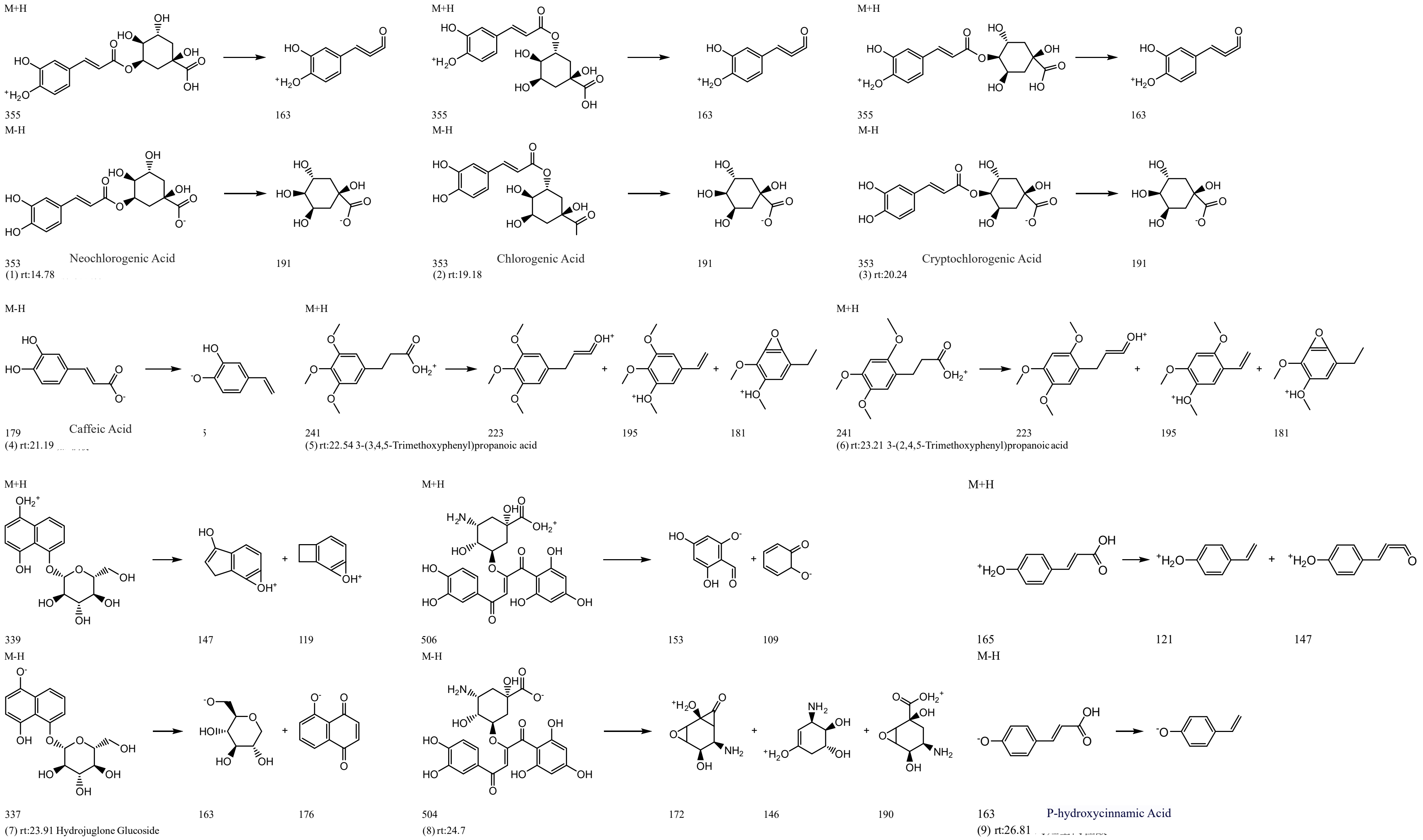

M+H

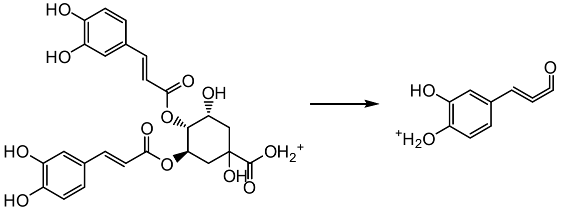

517 163

M-H

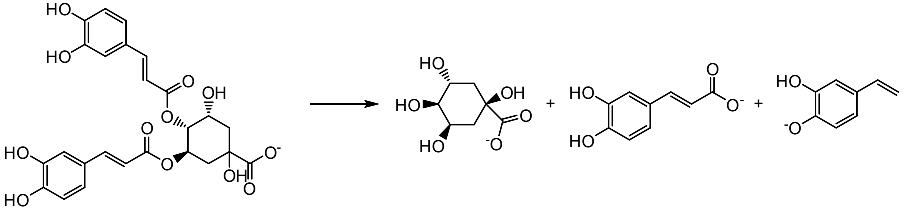

515 191 179 135

(10) rt:32.13 Isochlorogenic acid B

M+H

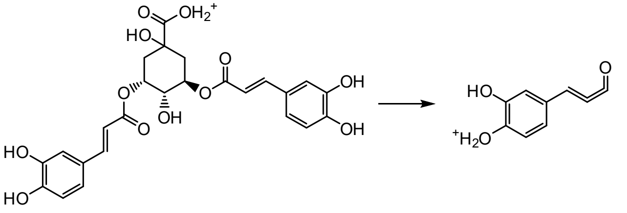

517 163

M-H

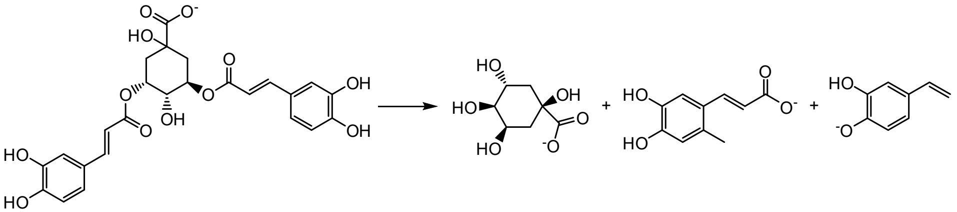

515 191 179 135

(12) rt:32.75 Isochlorogenic acid A

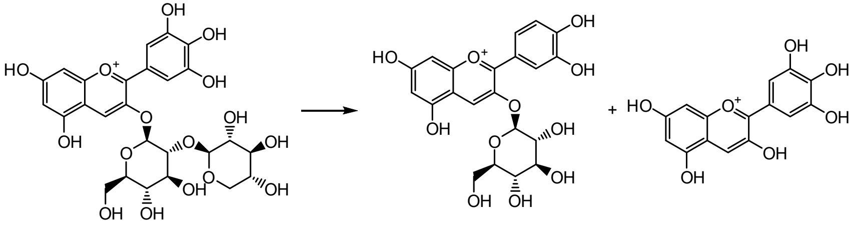

597 465 303

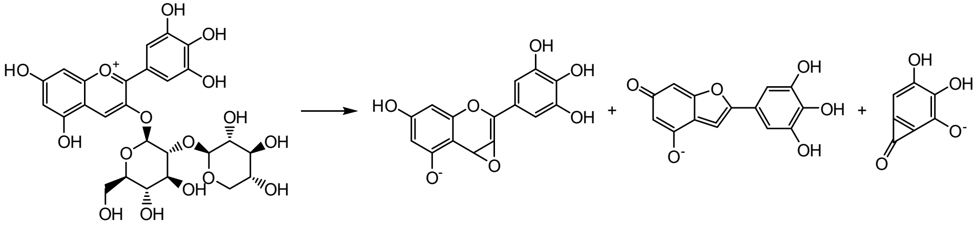

597 301 271 151

(11) rt:32.19 Delphinidin 3-sambubioside

M+H

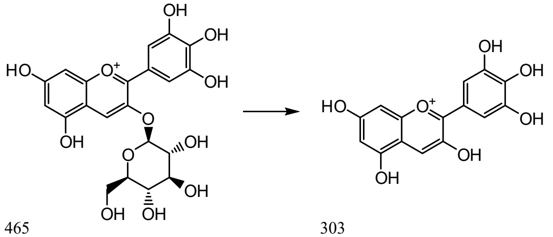

465 303

M-H

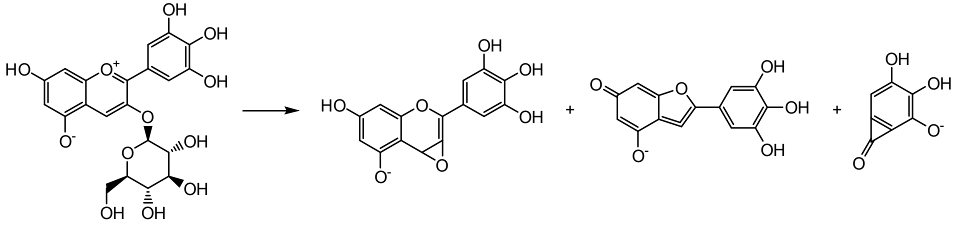

464 301 271 151

(13) rt:35.63 Delphinidin glucoside

M+H

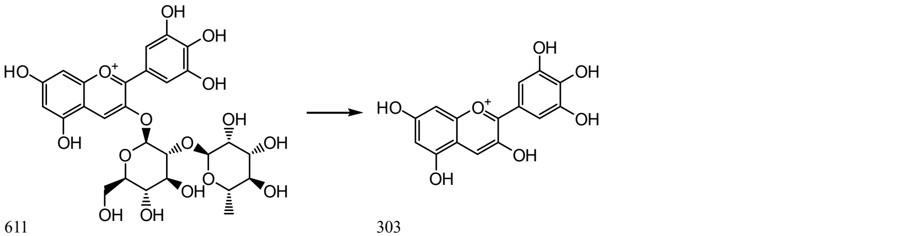

611 303

M-H

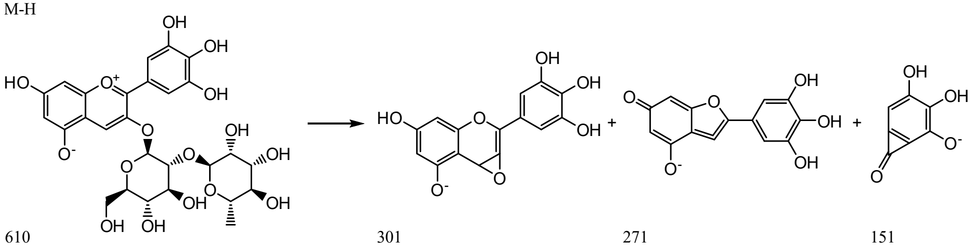

610 301 271 151

(14) rt:36.25

M+H

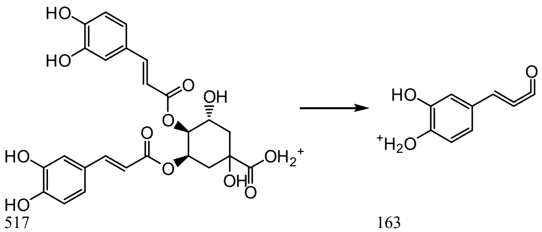

517 163

M-H

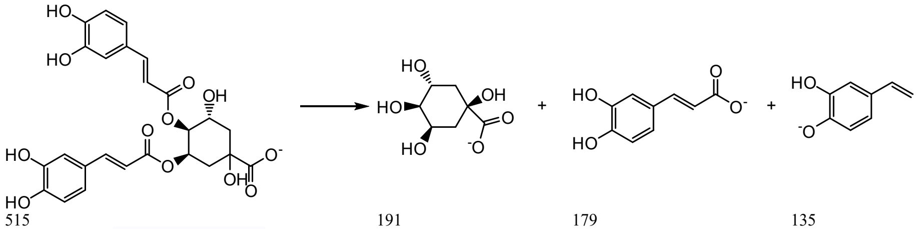

515 191 179 135

(15) rt:37.70 Isochlorogenic acid C

M+H

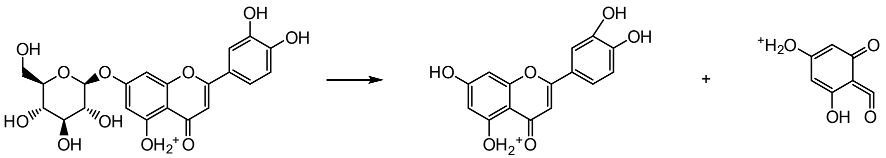

449 287 153

M-H

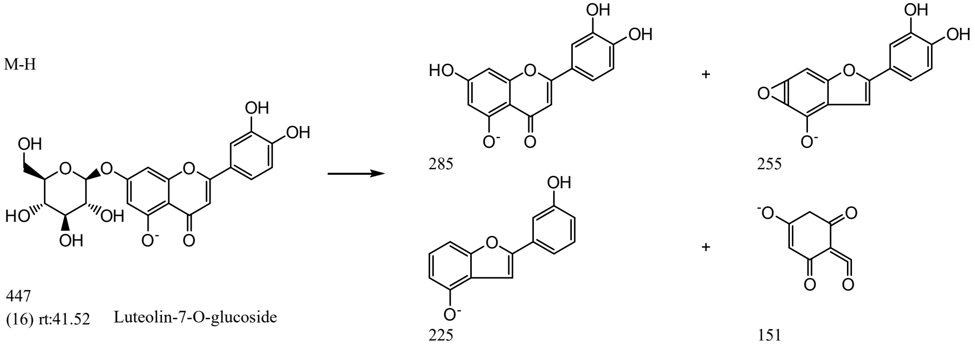

447

(16) rt:41.52 Luteolin-7-O-glucoside

M+H

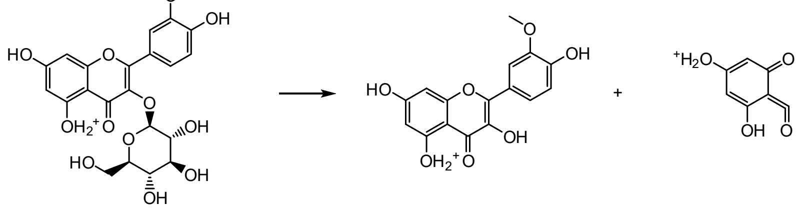

479 317 153

M-H

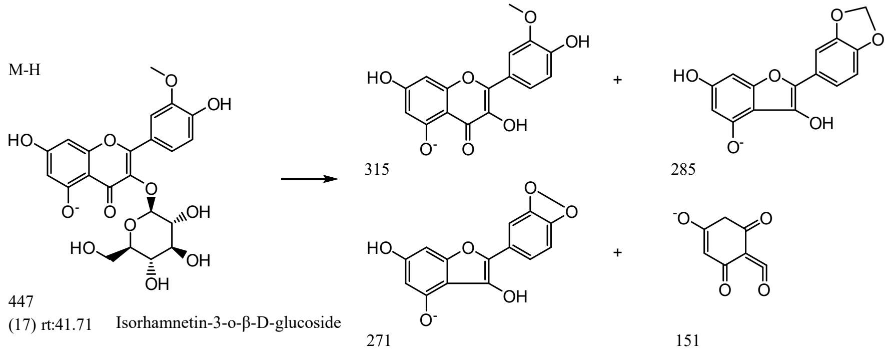

447

(17) rt:41.71 Isorhamnetin-3-o-β-D-glucoside

M+H

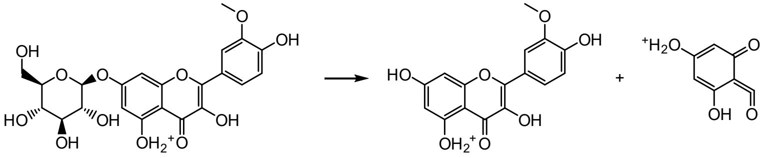

M+H

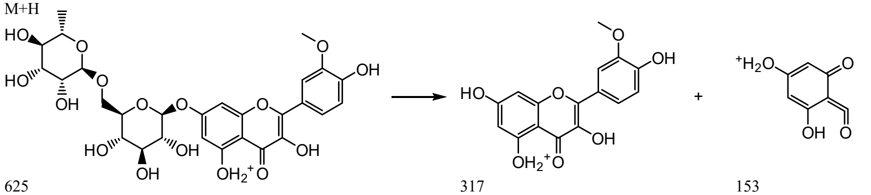

M-H

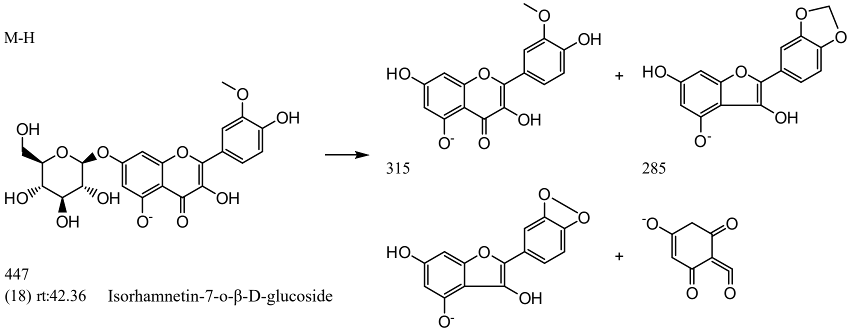

(18) rt:42.36 Isorhamnetin-7-o-β-D-glucoside

M-H

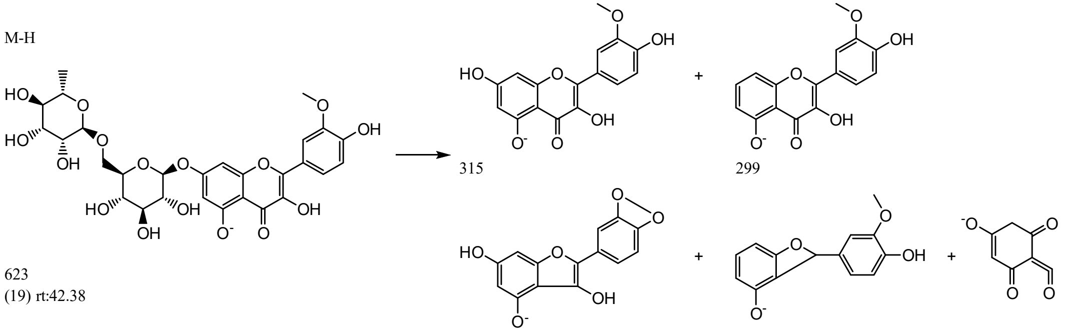

(19) rt:42.38

M+H

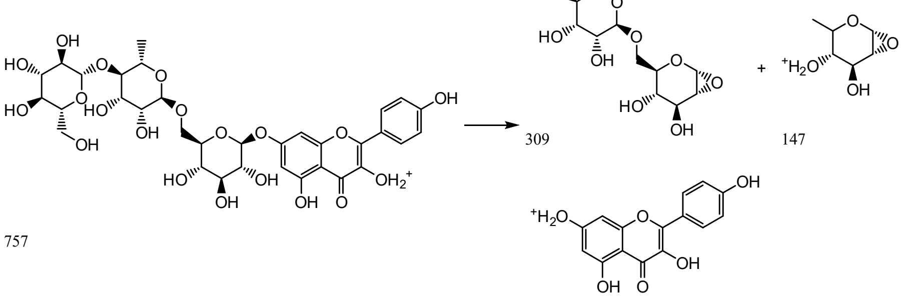

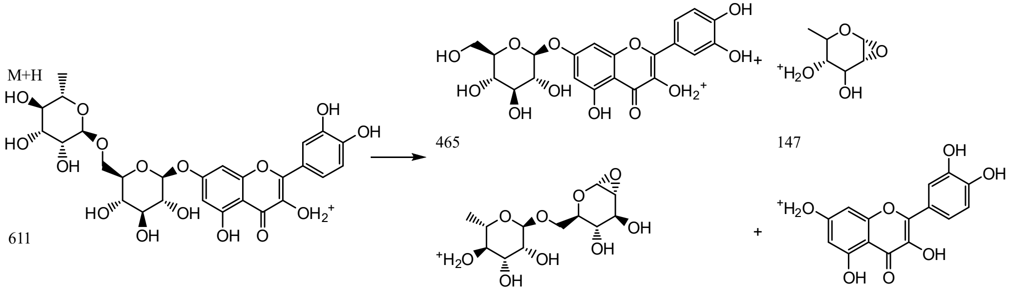

M-H

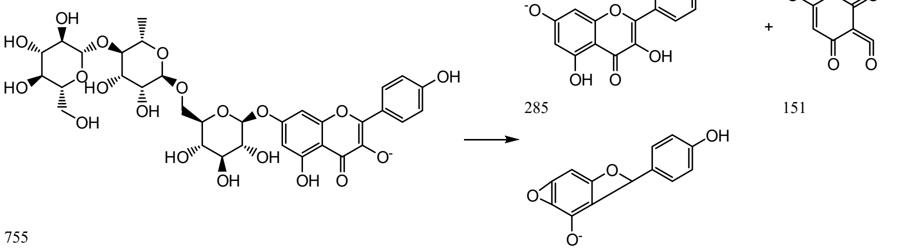

(20) rt:43.82

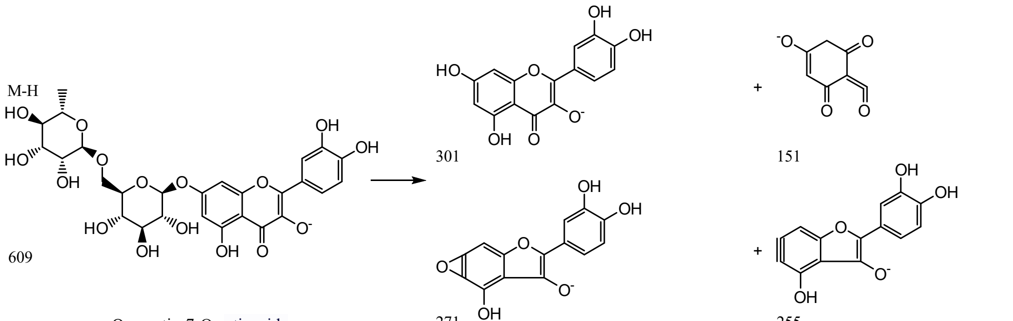

(21) rt:46.07 Quercetin-7-O-rutinoside

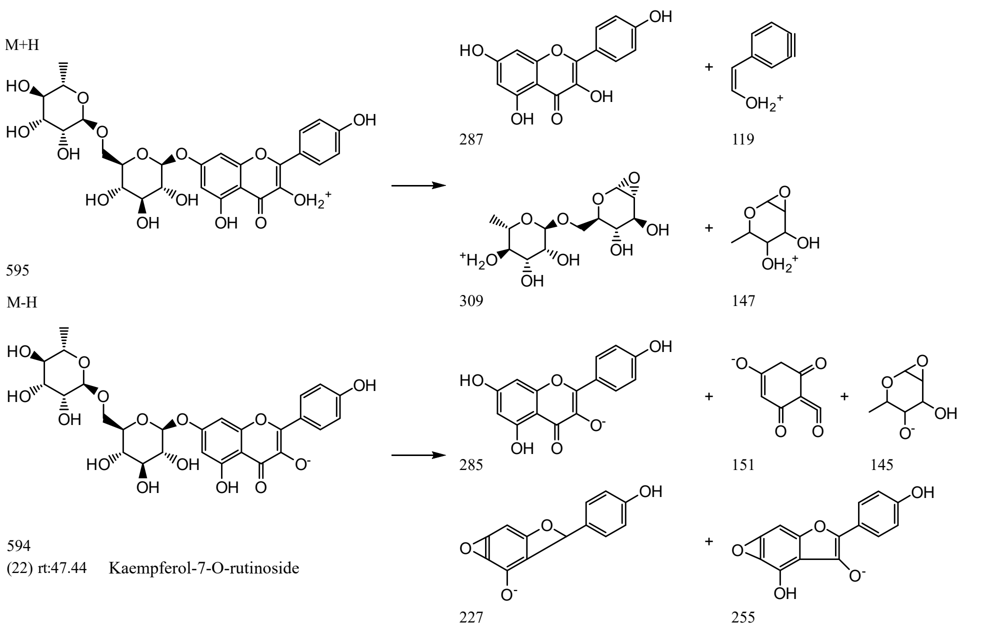

(22) rt:47.44 Kaempferol-7-O-rutinoside

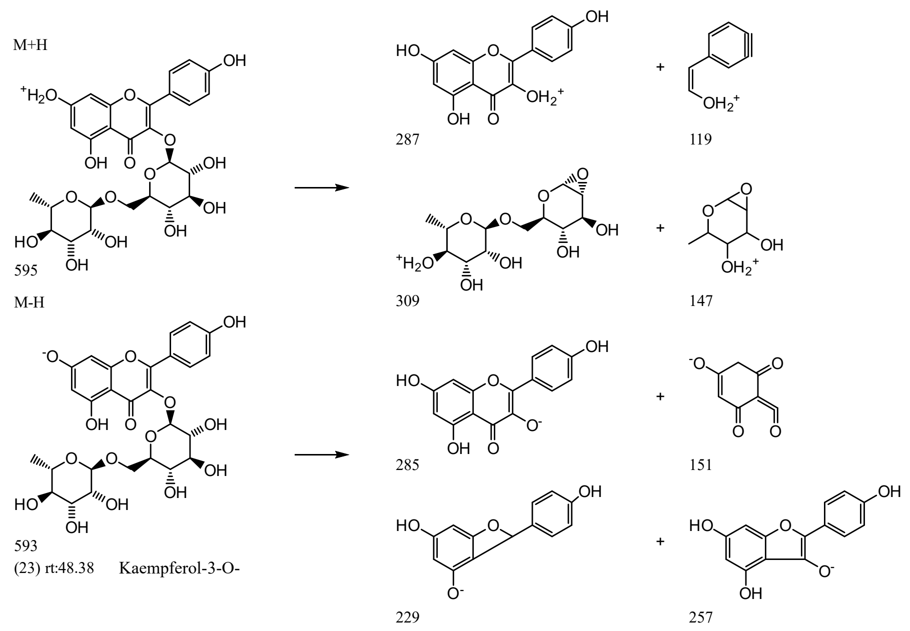

(23) rt:48.38 Kaempferol-3-O-

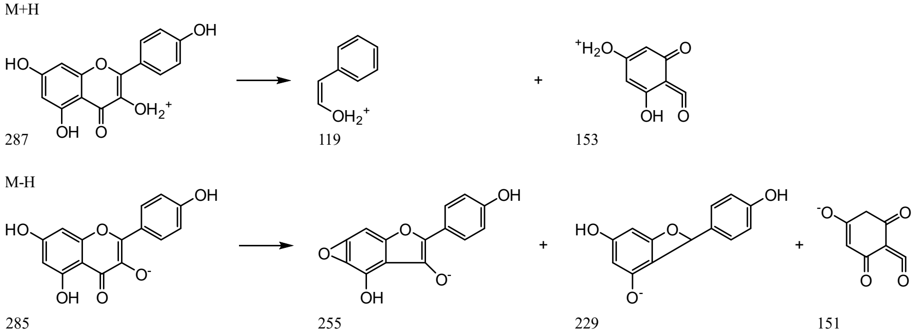

(24) rt:48.71 Kaempferol

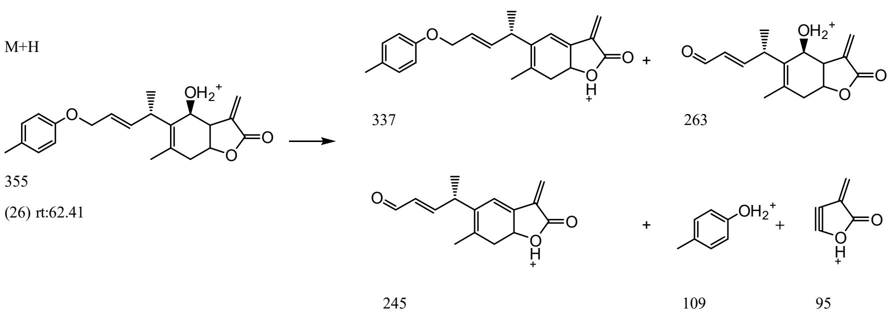

(26) rt:62.41

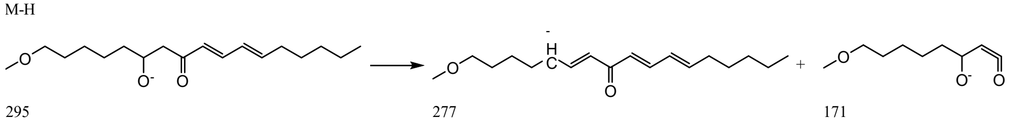

(25) rt:59.76

Table.S2 The results of the linear relationship for the five chemical components.

Chemical components	Regression equation	R^2	Linear Range
Chlorogenic acid	Y=7065.4X+25.382	0.9997	53.400~534.000
Esculin	Y=6701.7X+18.693	0.9990	14.100~141.000
1,4-Dicaffeoylquinic acid	Y=8669.1X-34.35	0.9998	12.450~124.500
Isochlorogenic acid A	Y=7224.1X+7.675	0.9993	13.500~135.000
1,5-Dicaffeoylquinic acid	Y=8154.4X+122.01	0.9995	171.000~1710.00

Table.S3 Determination results of 5 chemical components in 19 batches of *CS* samples.

Number	Chlorogenic acid	Esculin	1,4-Dicaffeoylquinic acid	Isochlorogenic acid A	1,5-Dicaffeoylquinic acid
	Mass percentage (mg/g)				
S1	0.9004	0.0348	0.0766	0.2234	3.3005
S2	0.7585	0.0831	0.1354	0.2211	3.2643
S3	0.9230	0.0572	0.1340	0.2231	3.4293
S4	0.7900	0.0889	0.1106	0.1956	3.1334
S5	0.4902	0.2410	0.0540	0.1047	1.4509
S6	0.7346	0.0441	0.1047	0.1717	2.6782
S7	1.0553	0.0477	0.1412	0.2372	3.6973
S8	0.9073	0.0449	0.1416	0.1932	3.4219
S9	0.8924	0.0558	0.1362	0.1893	3.3429
S10	1.0065	0.0686	0.1506	0.2096	3.6799
S11	0.6384	0.0616	0.0916	0.1801	2.6813
S12	1.1154	0.0434	0.1695	0.2715	4.3194
S13	0.7668	0.1149	0.1540	0.2371	3.3556
S14	0.6124	0.1245	0.0661	0.1548	1.4087
S15	0.7614	0.1596	0.1320	0.2178	2.5094
S16	0.8970	0.1445	0.1748	0.2272	2.8883
S17	1.1503	0.1060	0.1919	0.2077	3.9618
S18	1.1943	0.1255	0.2364	0.2118	4.1686
S19	0.8901	0.1853	0.1768	0.2454	3.0710

Table.S4 Distribution of Mid infrared spectral training and test sets in *CS* samples.

Datasets	Number of samples	Chlorogenic acid	Esculin	1,4-Dicaffeoylquinic acid	Isochlorogenic acid A	1,5-Dicaffeoylquinic acid
		Mass percentage (mg/g)				
Training set	15	0.4902~1.1943	0.0348~0.2410	0.0540~0.2364	0.1047~0.2773	1.4087~4.3194
Test set	4	0.6124~1.1154	0.0434~0.1596	0.0661~0.1769	0.1548~0.2372	2.5094~4.1686

Table.S5 Infrared model results of chlorogenic acid.

Chemical Components	Variable selection	Preorocessing	MIR								
			LVs	R^2cv	RMSECV	RPD_cv	RER_cv	R^2pre	RMSEP	RPD_pre	RER_pre
Chlorogenic Acid	Full spectrum	Raw	10	0.5066	0.1269	1.4236	5.5485	0.6960	0.1018	1.8137	4.9411
		S-G	10	0.5077	0.1267	1.4252	5.5572	0.6977	0.1015	1.8188	4.9557
		SNV	9	0.8024	0.0803	2.2496	8.7684	0.7353	0.0950	1.9437	5.2947
		MSC	9	0.8019	0.0804	2.2468	8.7575	0.7354	0.0950	1.9440	5.2947
	MW	Raw	5	0.8367	0.0730	2.4746	9.6452	0.8025	0.0821	2.2502	6.1267
		S-G	7	0.8355	0.0733	2.4656	9.6057	0.8012	0.0823	2.2428	6.1118
		SNV	7	0.7518	0.0900	2.0072	7.8233	0.7541	0.0916	2.0166	5.4913
		MSC	10	0.3756	0.0892	1.2655	7.8935	0.7315	0.0957	1.9299	5.2560
	UVE-SPA	Raw	8	0.9559	0.0379	4.7619	18.5778	0.9205	0.0520	3.5466	9.6731
		S-G	9	0.9548	0.0384	4.7036	18.3359	0.9345	0.0473	3.9073	10.6342
		SNV	5	0.9386	0.0447	4.0357	15.7517	0.7525	0.0919	2.0101	5.4733
		MSC	8	0.9309	0.0475	3.8042	14.8232	0.9082	0.0559	3.3005	8.9982
	ICO	Raw	8	0.9484	0.0396	4.4023	17.7803	0.5965	0.1173	1.5743	4.2882
		S-G	5	0.9507	0.0401	4.5038	17.5586	0.3133	0.1530	1.2067	3.2876
		SNV	4	0.9396	0.0444	4.0689	15.8581	0.8944	0.0600	3.0773	8.3833
		MSC	7	0.9339	0.0464	3.8895	15.1746	0.8987	0.0588	3.1419	8.5544

Table.S6 Infrared model results of Aesculin.

Chemical Components	Variable selection	Preorocessing	MIR							
			LVs	R^2cv	RMSECV	RPD_cv	RER_cv	R^2pre	RMSEP	RPD_pre	RER_pre
Aesculin	Full spectrum	Raw	10	-0.1212	0.0602	0.9444	3.4252	-0.0747	0.0473	0.9646	2.4567
		S-G	10	-0.1213	0.0602	0.9444	3.4252	-0.0746	0.0473	0.9647	2.4567
		SNV	10	-0.0626	0.0586	0.9701	3.5188	0.8422	0.0181	2.5174	6.4199
		MSC	10	-0.0628	0.0586	0.9700	3.5188	0.8420	0.0181	2.5158	6.4199
	MW	Raw	10	0.7483	0.0285	1.9932	7.2351	0.2511	0.0395	1.1555	2.9418
		S-G	6	0.8861	0.0192	2.9630	10.7396	0.3288	0.0374	1.2206	3.1070
		SNV	10	0.5852	0.0366	1.5527	5.6339	0.7668	0.0220	2.0708	5.2818
		MSC	10	0.5791	0.0369	1.5414	5.5881	0.7552	0.0226	2.0211	5.1416
	UVE-SPA	Raw	10	0.3261	0.0467	1.2182	4.4154	0.7750	0.0217	2.1082	5.3548
		S-G	10	0.7070	0.0308	1.8474	6.6948	0.7143	0.0244	1.8709	4.7623
		SNV	10	0.1500	0.0524	1.0847	3.9351	0.8046	0.0202	2.2622	5.7525
		MSC	10	0.8957	0.0184	3.0964	11.2065	-0.0058	0.0458	0.9971	2.5371
	ICO	Raw	10	0.6688	0.0327	1.7376	6.3058	-2.3600	0.0837	0.5455	1.3883
		S-G	10	0.6924	0.0315	1.8030	6.5460	-2.3557	0.0836	0.5459	1.3900
		SNV	7	0.9741	0.0092	6.2137	22.4130	-0.0066	0.0458	0.9967	2.5371
		MSC	4	0.9793	0.0082	6.9505	25.1463	-0.0463	0.0467	0.9776	2.4882

Table.S7 Infrared model results of 1,4-Dicaffeoylquinic acid.

Chemical Components	Variable selection	Preorocessing	MIR								
			LVs	R^2cv	RMSECV	RPD_cv	RER_cv	R^2pre	RMSEP	RPD_pre	RER_pre
1,4-Dicaffeoylquinic acid	Full spectrum	Raw	10	0.7357	0.0231	1.9451	7.8961	0.4135	0.0307	1.3058	3.9772
		S-G	10	0.7359	0.0231	1.9459	7.8961	0.4163	0.0306	1.3089	3.9902
		SNV	10	0.5360	0.0306	1.4681	5.9608	0.7322	0.0207	1.9324	5.8986
		MSC	10	0.5346	0.0306	1.4658	5.9608	0.7325	0.0207	1.9335	5.8986
	MW	Raw	10	0.5847	0.0289	1.5517	6.3114	0.4689	0.0292	1.3722	4.1815
		S-G	10	0.5858	0.0289	1.5538	6.3114	0.4658	0.0301	1.3682	4.0565
		SNV	10	0.7252	0.0235	1.9076	7.7617	0.3637	0.0319	1.2536	3.8276
		MSC	10	0.7212	0.0237	1.8939	7.6962	0.3571	0.0321	1.2472	3.8037
	UVE-SPA	Raw	5	0.8981	0.0143	3.1327	12.7552	0.3705	0.0318	1.2604	3.8396
		S-G	5	0.9403	0.0110	4.0927	16.5818	0.9070	0.0122	3.2791	10.0082
		SNV	8	0.8916	0.0148	3.0373	12.3243	0.9292	0.0107	3.7582	11.4112
		MSC	7	0.8913	0.0148	3.0331	12.3243	0.9730	0.0066	6.0858	18.5000
	ICO	Raw	6	0.9004	0.0142	3.1686	12.8451	0.5530	0.0268	1.4957	4.5560
		S-G	9	0.8353	0.0182	2.4641	10.0220	0.7560	0.0198	2.0244	6.1667
		SNV	8	0.8479	0.0175	2.5641	10.4229	-0.6117	0.0508	0.7877	2.4035
		MSC	7	0.8148	0.0193	2.3237	9.4508	0.8151	0.0172	2.3256	7.0988

Table.S8 Infrared model results of Isochlorogenic acid A.

Chemical Components	Variable selection	Preorocessing	MIR								
			LVs	R^2cv	RMSECV	RPD_cv	RER_cv	R^2pre	RMSEP	RPD_pre	RER_pre
Isochlorogenic acid A	Full spectrum	Raw	10	0.5869	0.0238	1.5559	7.2521	-0.1733	0.0337	0.9232	2.6706
		S-G	10	0.5872	0.0238	1.5564	7.2521	-0.1734	0.0337	0.9232	2.6706
		SNV	10	0.3706	0.0294	1.2605	5.8707	0.1127	0.0293	1.0616	3.0717
		MSC	10	0.3709	0.0294	1.2608	5.8707	0.1159	0.0293	1.0635	3.0717
	MW	Raw	10	0.0209	0.0366	1.0106	4.7158	0.2929	0.0262	1.1892	3.4351
		S-G	10	0.1454	0.0342	1.0817	5.0468	0.2056	0.0278	1.1220	3.2374
		SNV	4	0.8230	0.0156	2.3769	11.0641	-3.0588	0.0627	0.4964	1.4354
		MSC	4	0.8108	0.0161	2.2990	10.7205	-1.0457	0.0445	0.6992	2.0225
	UVE-SPA	Raw	7	0.9566	0.0077	4.8002	22.4156	0.5893	0.0200	1.5604	4.5000
		S-G	9	0.8298	0.0153	2.4239	11.2810	-0.1196	0.0329	0.9451	2.7356
		SNV	10	0.5203	0.0256	1.4438	6.7422	0.5904	0.0199	1.5625	4.5226
		MSC	4	0.9760	0.0057	6.4550	30.2807	0.3709	0.0247	1.2608	3.6437
	ICO	Raw	7	0.9463	0.0086	4.3153	20.0698	-0.2878	0.0353	0.8812	2.5496
		S-G	7	0.9453	0.0087	4.2757	19.8391	-0.2714	0.0351	0.8869	2.5641
		SNV	4	0.9295	0.0098	3.7662	17.6122	-0.2789	0.0352	0.8843	2.5568
		MSC	6	0.9277	0.0100	3.7190	17.2600	-0.1893	0.0340	0.9170	2.6471

Table.S9 Infrared model results of 1,5-Dicaffeoylquinic acid.

Chemical Components	Variable selection	Preorocessing	MIR								
			LVs	R2cv	RMSECV	RPD_cv	RER_cv	R2pre	RMSEP	RPD_pre	RER_pre
1,5-Dicaffeoylquinic acid	Full spectrum	Raw	10	0.6955	0.3805	1.8122	7.6497	0.9318	0.2426	3.8292	10.3500
		S-G	9	0.6967	0.3798	1.8158	7.6638	0.9318	0.2425	3.8292	10.3542
		SNV	10	0.5549	0.4601	1.4989	6.3262	0.9053	0.2858	3.2496	8.7855
		MSC	10	0.5534	0.4608	1.4964	6.3166	0.9063	0.2842	3.2669	8.8350
	MW	Raw	5	0.9130	0.2034	3.3903	14.3102	0.9857	0.1111	8.3624	22.6004
		S-G	8	0.9157	0.2003	3.4442	14.5317	0.9871	0.1053	8.8045	23.8452
		SNV	5	0.9180	0.1975	3.4922	14.7377	0.9512	0.2052	4.5268	12.2364
		MSC	8	0.9187	0.1966	3.5072	14.8052	0.9527	0.2020	4.5980	12.4302
	UVE-SPA	Raw	8	0.8684	0.2502	2.7566	11.6335	0.8743	0.3292	2.8205	7.6273
		S-G	9	0.9812	0.0947	7.2932	30.7360	0.9310	0.2440	3.8069	10.2906
		SNV	7	0.9568	0.1433	4.8113	20.3119	0.9667	0.1695	5.4800	14.8136
		MSC	4	0.9498	0.1544	4.4632	18.8517	0.9437	0.2203	4.2145	11.3976
	ICO	Raw	8	0.8721	0.2466	2.7962	11.8033	0.7843	0.4313	2.1532	5.8217
		S-G	10	0.2912	0.5806	1.1878	5.0133	0.7350	0.4781	1.9426	5.2518
		SNV	5	0.9569	0.1432	4.8168	20.3261	0.7995	0.4158	2.2333	6.0387
		MSC	6	0.9461	0.1601	4.3073	18.1805	0.6567	0.5441	1.7067	4.6148
